# Revealing circadian mechanisms of integration and resilience by visualizing clock proteins working in real time

**DOI:** 10.1038/s41467-018-05438-4

**Published:** 2018-08-14

**Authors:** Tetsuya Mori, Shogo Sugiyama, Mark Byrne, Carl Hirschie Johnson, Takayuki Uchihashi, Toshio Ando

**Affiliations:** 1 Department of Biological Sciences, Vanderbilt University, Nashville, TN 37235 USA; 2 Department of Physics, College of Science and Engineering, Kanazawa University, Kanazawa, 920-1192 Japan; 3 Department of Chemistry, Physics, and Engineering, Spring Hill College, 4000 Dauphin St., Mobile, AL 36608 USA; 4 Department of Molecular Physiology and Biophysics, Vanderbilt University School of Medicine, Nashville, TN 37232 USA; 5 Department of Physics and Structural Biology Research Center, Nagoya University, Chikusa-ku, Nagoya 464-8602 Japan; 6 Nano Life Science Institute (WPI-NanoLSI), Kanazawa University, Kanazawa, 920-1192 Japan

## Abstract

The circadian clock proteins KaiA, KaiB, and KaiC reconstitute a remarkable circa-24 h oscillation of KaiC phosphorylation that persists for many days in vitro. Here we use high-speed atomic force microscopy (HS-AFM) to visualize in real time and quantify the dynamic interactions of KaiA with KaiC on sub-second timescales. KaiA transiently interacts with KaiC, thereby stimulating KaiC autokinase activity. As KaiC becomes progressively more phosphorylated, KaiA’s affinity for KaiC weakens, revealing a feedback of KaiC phosphostatus back onto the KaiA-binding events. These non-equilibrium interactions integrate high-frequency binding and unbinding events, thereby refining the period of the longer term oscillations. Moreover, this differential affinity phenomenon broadens the range of Kai protein stoichiometries that allow rhythmicity, explaining how the oscillation is resilient in an in vivo milieu that includes noise. Therefore, robustness of rhythmicity on a 24-h scale is explainable by molecular events occurring on a scale of sub-seconds.

## Abstract (web summary)

The circadian clock proteins KaiA, KaiB, and KaiC reconstitute a circa-24 h oscillation of KaiC phosphorylation in vitro. Here the authors use high-speed atomic force microscopy to visualize in real time and quantify the dynamic interactions of KaiA with KaiC on the sub-second timescale to discover mechanisms of oscillatory resilience.

## Introduction

Circadian rhythms are circa-24 h oscillations in biological processes that are controlled by an endogenous biochemical pacemaker. The processes coordinated by these clocks range from gene expression, metabolism, and cell division to development and behavior^1^. A key diagnostic property of these biological clocks is the robust persistence of the oscillations in constant conditions (usually DD or LL at constant temperature). They must be noise resistant to combat the fluctuations of molecular components that can affect intracellular oscillators^2–4^. Synthetic cellular oscillators often produce distinctly irregular periodicities under different metabolic conditions^5,6^ and yet in contrast, circadian clocks are extremely precise^2,7^. The precision and resilience of these oscillators (± only a few min per day) is difficult to explain from biophysical and biochemical perspectives given their long 24-h timing circuit.

The quintessential example of biochemistry underlying circadian rhythms is the in vitro oscillator composed of the circadian proteins KaiA, KaiB, and KaiC from the model cyanobacterial system, *Synechococcus elongatus*^8–12^. These three proteins plus ATP reconstitute a circa-24 h rhythm of KaiC phosphorylation that robustly oscillates in vitro for at least 10 days without damping^13^. In addition to using ATP for its autokinase activity, KaiC hydrolyzes ATP and this reaction is an intrinsically constant-rate timer that drives KaiC phosphorylation and determines the period of the oscillation^14,15^. The status of KaiC phosphorylation feeds back to switch the KaiABC nanocomplex between its autokinase and autophosphatase states^11,16,17^. The KaiABC reaction uniquely remains the only circadian oscillator that has been reconstituted in vitro.

KaiC phosphorylation also oscillates in vivo^18,19^ with resilience under different conditions of environment, cell division status, and metabolism^4^. This remarkable characteristic is crucial for accurate timekeeping, but difficult to explain because KaiC phosphorylation in vitro oscillates within an allowed range of Kai protein stoichiometry^9^ and the concentrations of Kai proteins are likely to change under different metabolic/environmental conditions^3^. The transcription feedback loop is partly responsible for maintaining Kai proteins in appropriate concentrations and stoichiometry^4^, but a surprisingly broad range of permissive stoichiometry is also clear from analyses of the in vitro reaction^9,20,21^. What is not obvious, however, is the molecular mechanism(s) by which variations in Kai protein stoichiometry/concentration (that must occur in vivo) are tolerated so that consistent rhythmic amplitude and period is produced.

The process of testing a model for the intermolecular synchronization of KaiC phosphostatus in the in vitro KaiABC reaction^22^ led us to serendipitously discover dynamic high-frequency interactions between KaiA and KaiC that explain how a broader range of KaiA:KaiC stoichiometry is permissive. This result is unexpected because KaiA interacts with KaiC hexamers during the phosphorylation half-cycle phase of the in vitro oscillator repeatedly on and off on the timescale of fractions of seconds; this is a timescale that has been ignored in previous analyses of the KaiABC oscillator because interactions of this frequency are not intuitively expected to help explain a biochemical oscillation of circa-24 hours. These single-molecule methods reveal KaiC-phosphoform dependent differential affinity (PDDA) of KaiA for KaiC. PDDA enables the integration of high-frequency events into oscillations on the circadian timescale. Moreover, PDDA has broad consequences that were not anticipated by previous modeling and demonstrates an exquisite interplay of mechanisms that enable oscillatory resilience to changes in clock protein concentrations that are likely to occur in vivo^3,20^. We computationally and experimentally test PDDA-conferred resilience by in silico simulations and by biochemical manipulation of the in vitro KaiABC oscillation. These results indicate how a model biochemical oscillator is able to integrate high-frequency molecular events into a circadian timekeeper in environments of fluctuating clock protein levels resulting from molecular noise.

## Results

### Differential KaiA–KaiC affinity could stabilize oscillations

A hypothesis of allosteric control and differential affinity between KaiA and KaiC was proposed by van Zon and coworkers^22^ on the basis of a modeling approach with no specific experimental support to explain how KaiC hexamers maintain synchrony in the population of hexamers over the in vitro oscillation^13^. Briefly, they posited a differential affinity of KaiA for individual KaiC hexamers based on the state of KaiC’s phosphostatus that prevents the hexamers from drifting out of phase with each other during the half-cycle when KaiC is phosphorylating (Supplementary Fig. 1a–c)^22^. We will refer to this phenomenon as KaiC Phosphoform-Dependent Differential Affinity for KaiA (PDDA). To ascertain if PDDA can potentially explain synchronization in the phosphorylation half-cycle, we more fully explored the parameter space of the simplified van Zon model^22^, and confirmed that PDDA could stabilize oscillations (Supplementary Fig. 1d). Additionally, our in silico analyses of the van Zon et al. model predict only a limited range of variation in PDDA values that support oscillations; stronger PDDA values can result in KaiC population dephosphorylation due to insufficient KaiA association (large α) and PDDA values that are too weak do not sufficiently synchronize the population (small α, Supplementary Fig. 1d).

KaiA is a critical regulator of the remarkable switch of KaiC hexamers between autokinase and autophosphatase states^12,23–25^. At physiological temperatures, the autophosphatase activity of KaiC predominates when KaiA is absent. On the other hand, when KaiA is present, the KaiA homo-dimers enhance KaiC’s autokinase activity by transiently binding to the C-terminal peptide tentacles of KaiC during the phosphorylation phase^18,26^, triggering autophosphorylation of T432 and S431 residues within the KaiCII ring^27,28^ (or alternatively, by inhibiting dephosphorylation^29^). T432 is phosphorylated first in the cycle, followed by phosphorylation at S431 (S/T → S/pT → pS/pT)^16,30^. KaiB binds to hyperphosphorylated KaiC^30^ and the KaiB•C complex sequesters KaiA into a stable KaiA•B•C complex where both KaiA and KaiB are bound to the CI ring of KaiC so that KaiA is no longer available for stimulation of KaiC autophosphorylation^16,25,31^. Sequestration into the stable KaiA•B•C complex thwarts KaiA’s activity, enabling KaiC’s autophosphatase to take over and dephosphorylation of T432 and S431 proceeds via a phosphotransferase reaction that results in the regeneration of ATP from the phosphates at T432/S431 and the ADP bound at the P-loop^32,33^. The dephosphorylation reaction proceeds (pS/pT → pS/T → S/T) such that the overall process from the hypo- to the hyper- and back to the hypo-phosphorylated state of KaiC involves the following stages: S/T → S/pT → pS/pT → pS/T → S/T^16,30^. The dephosphoryation half-cycle is characterized as well by exchange of monomers among the hexamers in the population of KaiC molecules^9,13,34^. KaiA sequestration and KaiC monomer exchange aid inter-hexamer synchrony during the half-cycle in which KaiC is dephosphorylating (Supplementary Fig. 1a)^4,9,13,16,34–36^.

### Visualizing KaiC hexamers by single-molecule HS-AFM

**Fig. 1:**
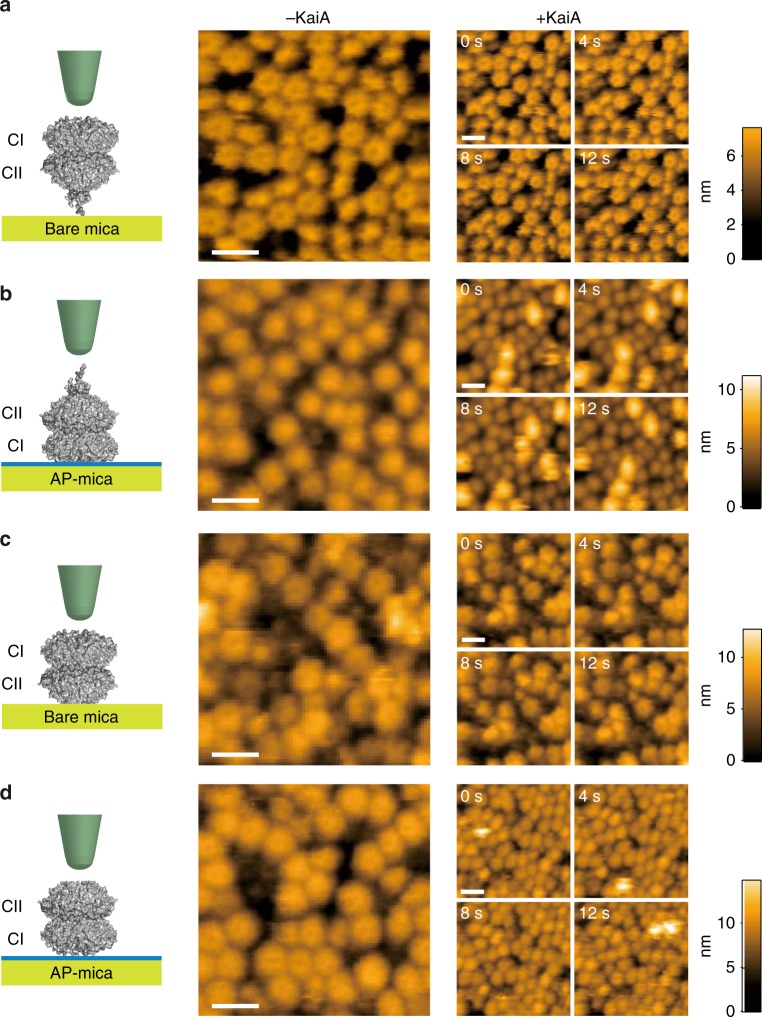
Orientation-specific immobilization of KaiC^WT^ and interaction with KaiA. **a** KaiC^WT^ on bare mica without (middle) and with (right) the addition of KaiA. **b** KaiC^WT^ on AP-mica without (middle) and with (right) the addition of KaiA. **c** KaiC with deletion of C-terminal tentacles (KaiC-ΔC) on bare mica without (middle) and with (right) the addition of KaiA. **d** KaiC with deletion of C-terminal tentacles (KaiC-ΔC) on AP-mica without (middle) and with (right) the addition of KaiA. All images shown in the middle and the right columns were acquired at a frame rate of 1 fps and 1.25 fps, respectively. The images on the right columns are shown every 4 s. Scale bar = 15 nm. Also see Supplementary Movies 1–4

To test the PDDA model of hexamer synchronization during the half-cycle when KaiC is phosphorylating^22^, we set out to measure the phase-dependent dynamics of interaction of individual KaiA–KaiC molecules, which occur on the timescale of seconds or less^9,21,31,37^. Among biophysical techniques to study protein–protein interactions at the single molecule level, high-speed atomic force microscopy (HS-AFM) is unique in its ability to directly observe individual proteins at submolecular spatial resolution and sub-100 ms time resolution in physiological solutions^38–40^ and is therefore ideal for this test. Figure 1 shows that orientation-specific immobilization of native wild-type KaiC hexamers (KaiC^WT^) was achieved on surfaces of either bare mica or 3-aminopropyl-trietoxy silane-treated mica (AP-mica). KaiC bound to AP-mica was available to interact with KaiA (Fig. 1b), but KaiC in the opposite orientation as bound to bare mica did not interact with KaiA (Fig. 1a), nor was KaiC with deletion of the C-terminal tentacles able to interact with KaiA (Fig. 1c, d). The orientation of KaiC binding to bare mica versus AP-mica is probably due to the major differences in the electrostatic charges on the opposite poles of KaiC hexamers; in particular, the CI pole has mostly negative charge, while the CII pole has mostly positive charge^41^.

**Fig. 2:**
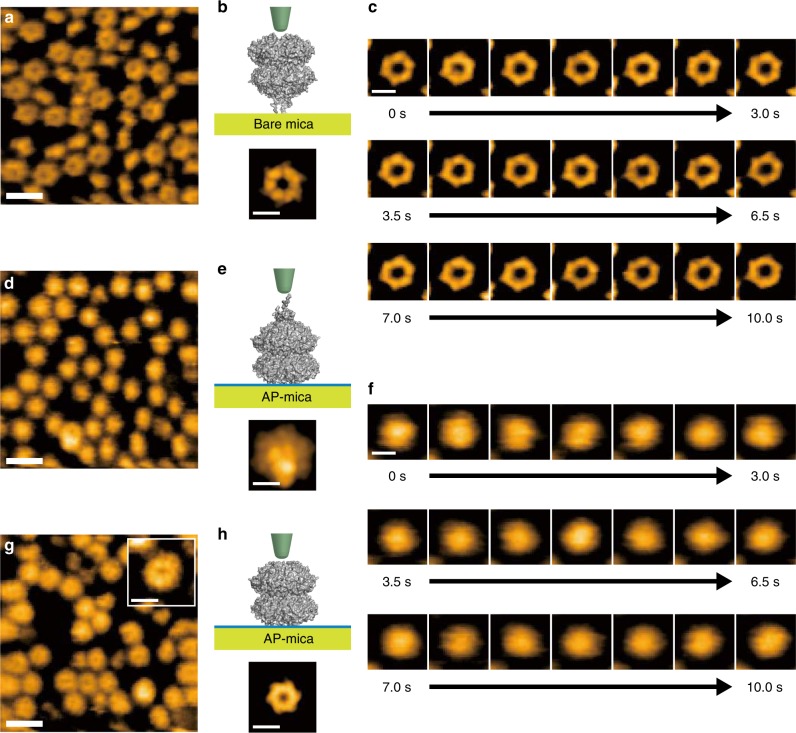
HS-AFM images of KaiC^WT^ hexamers. **a** HS-AFM images of native KaiC^WT^ hexamers immobilized on bare mica. Scale bar = 15 nm. **b** Simulated AFM image of KaiC (bottom) viewed from the N-terminal side. This construction was made using a hypothetical probe with a radius of 0.5 nm and a cone angle of 10°, as illustrated (top). Scale bar = 5 nm. **c** Time lapse images of KaiC^WT^ hexamers attached to bare mica, which visualizes the N-terminal side of KaiC hexamers in the presence of 2 mM Mg^2+^-ATP. Images were acquired at a frame rate of 2 fps and images taken every 0.5 s are shown. Scale bar = 5 nm. **d** HS-AFM image of native KaiC^WT^ hexamers immobilized on AP-mica. Scale bar = 15 nm. **e** Simulated AFM image of KaiC (bottom) viewed from the C-terminal side. This construction was made using a hypothetical probe with a radius of 0.5 nm and a cone angle of 10°, as illustrated (top). Scale bar = 5 nm. **f** Time lapse images of KaiC hexamers attached to AP-mica. The images show the C-terminal side of KaiC^WT^ hexamers in the presence of 2 mM Mg^2+^-ATP. Imaging was carried out at a frame rate of 2 fps and images every 0.5 s are shown. Scale bar = 5 nm. **g** HS-AFM images of KaiC-ΔC hexamers (in which the C-terminal tentacles are truncated), immobilized on bare mica. Scale bar = 15 nm. The insert is an enlarged image (scale bar within insert = 5 nm). **h** Simulated AFM image of KaiC-ΔC (bottom) viewed from the C-terminal side without the tentacles. This construction was made using a hypothetical probe with a radius of 0.5 nm and a cone angle of 10°, as illustrated (top). Also see Supplementary Movies 5–12

Images of the KaiC hexamers attached to a bare mica surface showed hexagonal ring-like structures with a pore in the center (Figs. 1a and 2a, c). Based on the crystal structure of KaiC^28,41^, a simulated AFM image of the N-terminal side of the hexamer (KaiCI) has a pore in the center (Fig. 2b) and matched the observed AFM image of the KaiC sample attached to bare mica (Fig. 2a, c). On the other hand, KaiC attached to AP-mica surfaces had no obvious pore in the center (Figs. 1b, 2d, f). The simulated AFM image of the C-terminal side of the hexamer (KaiCII) lacked a pore due to the presence of C-terminal tentacles that obscure the pore (Fig. 2e)^28,41^ and closely resembles the observed AFM image of KaiC hexamers attached to an AP-mica surface (Fig. 2d, f). Moreover, when the C-terminal tentacles are truncated (KaiC-ΔC), the pore in AP-mica-immobilized KaiC becomes apparent again (Fig. 2g, h). Figures 1 and 2 indicate that HS-AFM accurately detects the various orientations of KaiC, and that the N-terminal side of KaiC hexamer selectively attaches to AP-mica surfaces, thereby exposing the C-terminal tentacles of the KaiC hexamer (KaiCII) with which KaiA interacts and influences the conformation of the internal A-loop of KaiC^27,31,42^.

Since KaiC forms a hexamer^43,44^ and exhibits ATPase activity^14,29^, changes of conformation and subunit assembly within KaiC hexamers might be predicted to occur as a result of ATP hydrolysis and nucleotide exchange as previously observed by HS-AFM investigations of F_1_-ATPase^45^. Indeed, modest conformational changes due to phosphorylation and ATP hydrolyses have also been reported for the KaiC hexamer^15,46^. We monitored hyper- and hypo-phosphorylated KaiC hexamers in the imaging buffer containing Mg^2+^-ATP continuously in either the CI end-up (Fig. 2c) or CII end-up (Fig. 2f) orientations. However, we did not observe distortions of KaiC structure, conformational changes, or rotational subunit rearrangements within our observation time windows (Fig. 2c, f). Further high-resolution images of KaiC from the CI side confirmed the absence of rotational changes in conformation (Supplementary Fig. 2). Perhaps the fact that KaiC has remarkably low rates of (i) ATP hydrolysis (~90 ATP molecules hydrolyzed per day per KaiC hexamer)^14,15^ and (ii) autokinase/autophosphatase activities^18,23,29^ means that rotational conformational changes of KaiC are either too slow or too small to be detected by HS-AFM within our observation intervals.

### KaiA interaction depends upon KaiC phosphostatus

To explore the dynamics of the interaction of KaiA with KaiC, we immobilized native KaiC on an AP-mica surface and confirmed by HS-AFM the CII-up orientation of the hexamers. Then, native KaiA was added to the imaging solution, after which time an object (approximately 3.5-nm high) was transiently attracted to the C-terminal end of KaiC (‘+KaiA’ in Fig. 1b). Several lines of evidence confirm that this object was KaiA, which is known to interact with the C-terminal tentacles of KaiC^27^. As mentioned above, the bright object did not interact with KaiC^WT^ attached to a bare-mica surface in the CI-up orientation in which the C-terminal tentacles are unavailable for KaiA binding (Fig. 1a), nor did it interact with a mutated KaiC in which the KaiA-interacting tentacles are removed (aka KaiC-ΔC, Fig. 1c, d)^31,42^. Moreover, non-KaiA proteins such as bovine serum albumin (BSA) or green fluorescent protein (GFP) did not interact with the KaiC^WT^ hexamer (Supplementary Fig. 3). Therefore, the interaction is specific to KaiA and the tentacles of KaiCII^WT^, confirming that the bright 3.5-nm high object was KaiA.

**Fig. 3:**
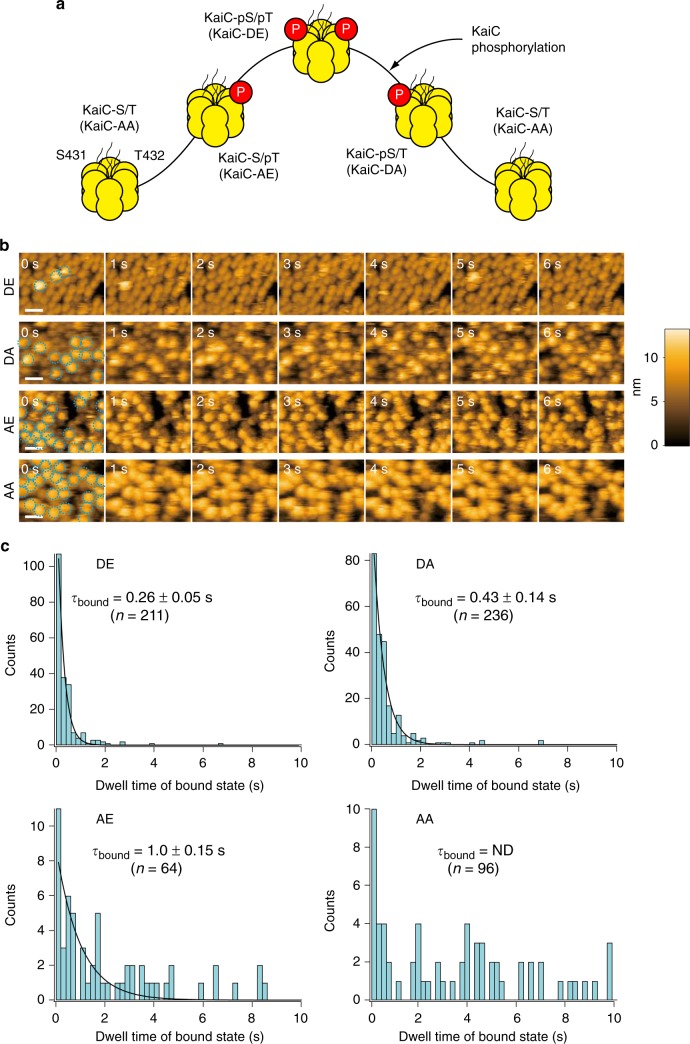
KaiC phospho-status modulates the binding affinity of KaiA for KaiC. **a** Circadian cycling of the phosphorylation state of KaiC. KaiC harbors two phosphorylation sites: S431 and T432 that undergo the sequential phosphorylation order shown over the circadian cycle^16,30^, where ‘p’ means the site is phosphorylated (e.g., ‘S/pT’ means that T432 is phosphorylated, but S431 is not). The phosphomimics for each stage in the phosphorylation cycle are shown in parentheses (e.g., ‘KaiC-AE’ is the phosphomimic for the ‘S/pT’ phosphoform of KaiC). **b** Various phospho-mimics of KaiC were attached to AP-mica in the CII-end up (C-terminal-up) orientation. KaiA was added to the observation solution and then the dynamic interaction between KaiA and the KaiC phospho-mimics was monitored by HS-AFM in the absence of ATP. The following phospho-mimics were used: DE, KaiC-DE (mimic of pS/pT); DA, KaiC-DA (mimic of pS/T); AE, KaiC-AE (mimic of S/pT), and KaiC-AA (mimic of S/T). Concentration of KaiA, 1 μM. Frame rate, 1 fps. Scale bars = 20 nm. Also see Supplementary Movies 13–16. **c** Dwell time analysis for KaiA-bound state of phospho-mimics of KaiC observed by HS-AFM. The AFM images used in this analysis were captured at 10 fps. Each black solid line overlaid on the corresponding histogram of dwell time was obtained by fitting the histogram to a first order reaction model with a time constant (τ_bound_) shown, except for the case of AA, where the value of τ_bound_ could not be determined (ND)

Mimics of the various phospho-states of KaiC are a valuable tool for characterizing the phosphorylation-dependent properties of KaiC^19,21,24,30,31^. HS-AFM measurements show dynamic KaiA–KaiC interactions at a single-molecule level that vary among the KaiC phospho-mimics (Fig. 3). The KaiA•KaiC complex formed frequently and dissociated within seconds for most of the KaiC phospho-mimics (Fig. 3b). (The data of Fig. 3b also support the conclusion that the phosphorylation state of the KaiC^WT^ hexamers did not alter the characteristics of KaiC^WT^ attachment to mica-based surfaces.) To determine the frequency of the binding reaction between KaiA and KaiC, the dwell times of the bound state of KaiC (on-event duration) were measured and are plotted in Fig. 3c. Consistent with the PDDA hypothesis, mimics of hypo-phosphorylated KaiC (KaiC-AE and KaiC-AA) bind KaiA for a significantly longer time (AE: τ_bound_ = 1.00 ± 0.15 s, AA: too slow to determine accurately) than mimics of hyperphosphorylated KaiC (DE: τ_bound_ = 0.26 ± 0.05 s, DA: τ_bound_ = 0.43 ± 0.14 s, Fig. 3c). When 1 mM ATP was included in the observation buffer, the τ_bound_ values were essentially identical: τ_bound_ for AE = 0.95 ± 0.04 s, DE = 0.31 ± 0.01 s, DA = 0.43 ± 0.01 s, and again τ_bound_ for AA was too slow to determine accurately (Supplementary Fig. 4).

### Cyclic KaiA–KaiC affinity during the in vitro oscillation

**Fig. 4:**
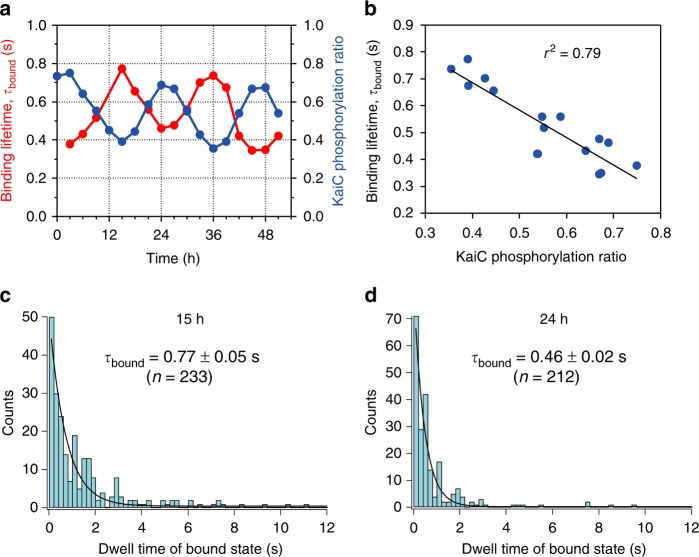
KaiA’s affinity to KaiC oscillates with the KaiC phosphorylation rhythm in vitro. **a** KaiA’s bound state lifetime (τ_bound_) depends on KaiC^WT^ phosphostatus over a 51-h time course of the in vitro cycle of phosphorylation. Purified KaiA (to a final concentration of 1.9 μM) was added to KaiC immobilized onto AP-mica and dynamics were observed by HS-AFM. See Supplementary Fig. 5 for dwell time data at each phase. Parallel samples were collected and immunoblotted to confirm KaiC phospho-status (blue) at each timepoint (Supplementary Fig. 6). KaiA–KaiC binding lifetime τ_bound_ was calculated from the bound-state dwell time analysis (red). **b** Correlation between the extent of KaiC phosphorylation and KaiA’s bound state lifetime (τ_bound_). **c**, **d** Dwell time analysis of KaiA’s bound state lifetime **c** at the peak phase of phosphorylation cycle (24-h timepoint in **a**) or **d** at the trough phosphorylation phase (15-h timepoint in **a**). Dwell time analysis at each time point is shown in Supplementary Fig. 5, and a replicate experiment is shown in Supplementary Fig. 7

The τ_bound_ values of the KaiC phosphomimics correlate with the circadian phase-dependent changes of KaiC^WT^ phosphostatus (Fig. 3a). Therefore, if the KaiC phosphomimics accurately reflect the various KaiC phospho-states, the data of Fig. 3 predict that KaiA–KaiC^WT^ affinity should oscillate over the circadian timescale in the in vitro KaiABC reaction due to the oscillation of KaiC phosphorylation status. In this in vitro oscillator, the three Kai proteins interact to form phase-dependent complexes^9^ such that during the phosphorylation phase, KaiA•KaiC complexes and unbound free KaiC hexamers predominate^34^. To determine experimentally whether the KaiA-binding affinity to native KaiC^WT^ changes over the in vitro oscillation, we observed the interaction of KaiA with KaiC^WT^ samples that had been removed at different phases from the in vitro reaction and applied to AP-mica (Fig. 4a). Free KaiA was added to the lawn of immobilized KaiC^WT^ hexamers to quantify the duration of the transient KaiA–KaiC interactions. As shown in Fig. 4a (and see also Supplementary Figs. 5-7), the affinity between KaiC and KaiA reproducibly oscillated in antiphase concert with the KaiC phosphorylation cycle. KaiC^WT^ from the peak phase of the phosphorylation cycle exhibited a lower average affinity to KaiA (τ_bound_ = 0.46 ± 0.02 s at the 24-h timepoint) while KaiC^WT^ from the trough phosphorylation phase exhibited a higher average affinity (τ_bound_ = 0.77 ± 0.05 s at 15 h)(Fig. 4a, c, d and Supplementary Fig. 5). These affinity values are averages due to the varying proportions of KaiC^WT^ phosphostates that are present at each phase of the in vitro oscillation. There is a strong correlation between the KaiC phosphorylation state and the mean lifetime of the KaiA•KaiC complex (Fig. 4b), namely that KaiA dwells for a longer time on unphosphorylated KaiC than on phosphorylated KaiC, as predicted from the PDDA hypothesis.

### KaiC phospho-status affects both *k*_off_ and *k*_on_ rates

**Fig. 5:**
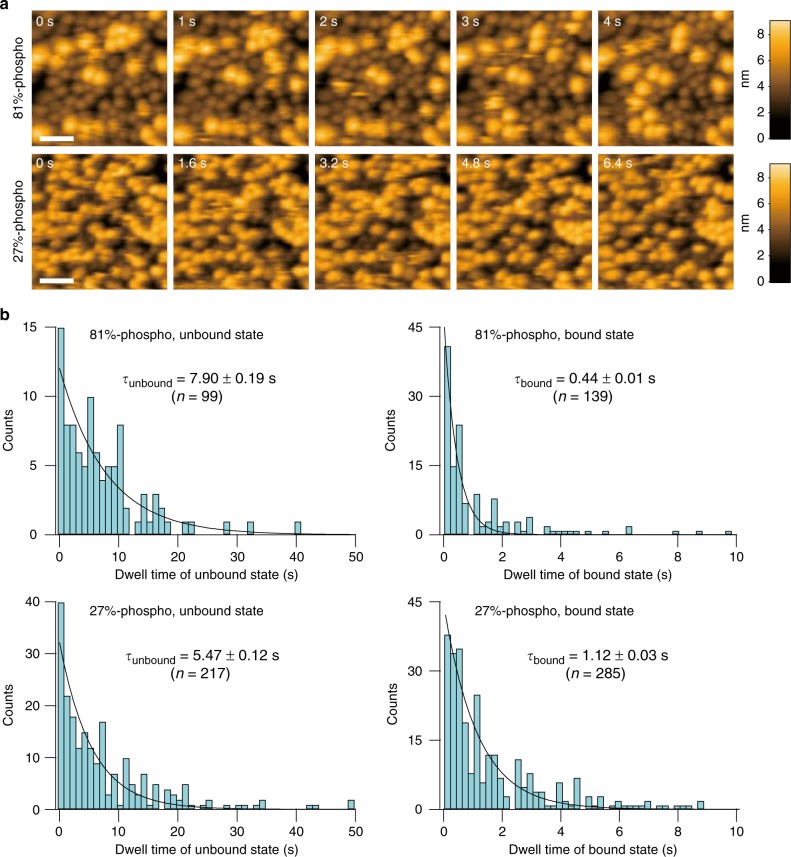
The values of τ_bound_ and τ_unbound_ depend upon KaiC’s phospho-status. **a** HS-AFM images of KaiC and KaiA interaction. Native KaiC hexamers (KaiC^WT^) that were approximately 81% phosphorylated (upper panels) or approximately 27% phosphorylated (lower panels) were immobilized on AP-mica surfaces and KaiA was added (final concentration of 0.4 µM) to the observation buffer. Images were acquired at a frame rate of 1 fps (upper) and 1.25 fps (lower). Brighter spots show KaiA bound to surface-immobilized KaiC. Scale bars = 30 nm. Also see Supplementary Movies 17, 18. **b** Dwell time analysis for KaiA-bound and KaiA-unbound states of hyperphosphorylated (81%; top) and hypo-phosphorylated (27%; bottom) KaiC. Each black line overlaid on the corresponding histogram of dwell time was obtained by fitting the histogram to a first order reaction model with a time constant (τ_bound_ or τ_unbound_) shown. ‘*n*’ shown in each graph indicates the number of detected events used for the analysis

To refine the salient characteristics of the hyperphosphorylated versus hypo-phosphorylated states of KaiC, we compared a KaiC^WT^ preparation that was 81% phosphorylated with a KaiC preparation that was 27% phosphorylated (Fig. 5). KaiA remains bound dramatically longer to hypo-phosphorylated KaiC^WT^ than to hyperphosphorylated KaiC^WT^; the bound-state dwell times (exponential time constant τ_bound_) for hypo-phosphorylated versus hyperphosphorylated KaiC^WT^ were 1.12 ± 0.03 s and 0.44 ± 0.01 s, respectively (Fig. 5b). We also estimated the unbound-state dwell times (τ_unbound_) from the interval of time in which KaiC hexamers remain unassociated between consecutive KaiA-binding events (Fig. 5b). Calculated unbound-state dwell times (τ_unbound_) for hypo- and hyperphosphorylated KaiC^WT^ were 5.47 ± 0.12 s and 7.90 ± 0.19 s, respectively. Equivalent results were obtained when the observation buffer contained ATP (Supplementary Fig. 8).

KaiA interactions with hypo- and hyperphosphorylated KaiC^WT^ (Figs. 4, 5) and hypo-phospho- and hyper-phospho-mimic KaiC derivatives (Fig. 3), conclusively demonstrate that initiation of KaiA–KaiC binding differs as a function of the phosphorylation state of KaiC hexamer (τ_unbound_) and once KaiA and KaiC are associated, enhanced KaiC phosphorylation destabilizes the association (τ_bound_). Therefore, the KaiC hexamer has differential affinity to KaiA depending on its phosphorylation state. Because KaiA binding to the C-terminal tentacles stimulates KaiC autokinase activity^27,42^, these differential KaiA–KaiC affinities (PDDA) undoubtedly contribute to KaiA-dependent differences in the rate of KaiC autophosphorylation as a function of KaiC phospho-status^21^.

### PDDA enhances oscillatory resilience

The consequences of these insights into KaiA–KaiC interactions were simulated with a constrained Monte Carlo model of population hexamer dynamics that was updated to include phosphoform transitions in each KaiC protomer (See Supplementary Methods for model details and parameters). The stochastic simulations simultaneously include several hundred distinct hexamer states in simple mathematical matrix form (including bound and unbound states of KaiC) and these simulations include intrinsic variability in the transition rates from Monte Carlo reaction probabilities. In contrast, a continuum ordinary differential equation (ODE) population model would need to include all the unique KaiC states of the system explicitly encoded in several hundred coupled ODEs (including multi-protein complexes) unless additional assumptions were made to lower the dimensionality of the system. In stochastic simulations PDDA was simulated by directly varying the relative binding probability of KaiA to KaiC as a function of KaiC hexamer phosphoform composition, while in non-PDDA simulations the relative binding probability of KaiA for KaiC was locked to a fixed value independent of hexamer composition. The simulations used a pseudo-steady-state calculation of the fraction of KaiA•KaiC complexes that form from a simple 1:1 binding model, A_2_+C_6_↔(A_2_C_6_). +PDDA was implemented by varying the dissociation of KaiA from KaiA•KaiC as a weighted function of the phospho-states based on the τ_unbound_ and τ_bound_ values (i.e., association rate *k*_on_ and dissociation rate *k*_off_) that were experimentally measured by HS-AFM (using the data from the phosphomimics for each phospho-state). Simulations of the KaiA–KaiC interactions indicate that +PDDA increases the fraction of pS/T states that form as compared with −PDDA, as expected from reduced KaiA binding to hyperphosphorylated states (Supplementary Figs. 9b, 10a). Intuitively, because KaiA becomes less effective in stimulating KaiC phosphorylation as the hexamers more closely approach the hyperphosphorylated pS/pT state, the dephosphorylation of hyperphosphorylated pS/pT to the pS/T phosphoform is facilitated.

**Fig. 6:**
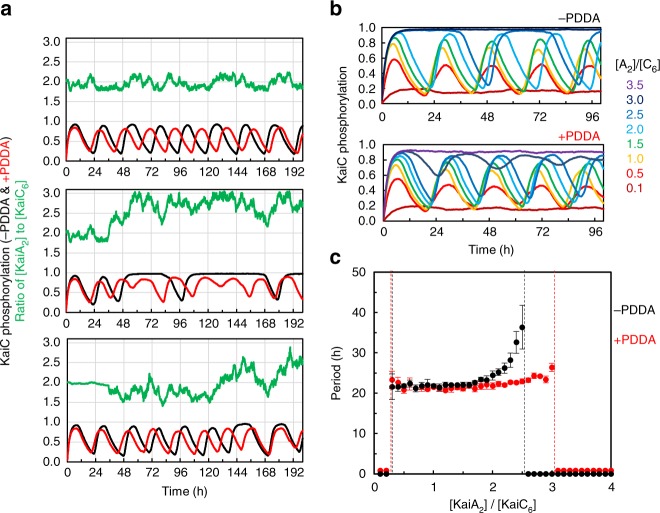
PDDA enhances resilience to variations in Kai protein stoichiometry. **a** Simulated effect of PDDA on oscillatory consistency. Three examples of simulated randomly changing variation of KaiA concentration (green) on KaiC phosphorylation patterns +PDDA (red) versus −PDDA (black). Concentrations of KaiC and KaiB were held constant as [KaiA] was varied. **b** Representative KaiC phosphorylation as the ratio of concentrations of KaiA dimer to KaiC hexamer was varied from the beginning of the time course without subsequent changes in KaiA, KaiB, or KaiC concentrations. Simulations were performed in the absence of PDDA (−PDDA, top) and with PDDA activated (+PDDA, bottom). The scale of [KaiA]: [KaiC] is shown to the right ([A_2_]/[C_6_]). In the case of −PDDA, the top two simulations ([A_2_]/[C_6_] = 3.0 and 3.5) coincide. **c** Range of allowed oscillatory regime (dashed vertical lines) and estimated oscillatory period as KaiA dimer to KaiC hexamer ratio ([KaiA_2_]/[KaiC_6_]) was varied with (+PDDA, red) or without PDDA (−PDDA, black). Simulations performed as shown in **b**. Absence of oscillations is indicated as period = 0. Error bars are ±S.D.

Our simulations were first directed toward testing the synchronization effect predicted by van Zon and coworkers^22^. Unexpectedly, the magnitude of PDDA that our experiments verified did not support the original predictions upon which we originally undertook this project. Our HS-AFM measurements indicated an approximate 10 × change in KaiA *k*_on_ and *k*_off_ rates between hyper- and hypo-phosphorylated KaiC, but the synchronization effect predicted by van Zon and coworkers in their modeling study^22^ suggested that ~100 × −1000 × changes in PDDA are needed for sustained oscillations (see Supplementary Table 2 of van Zon et al., 2007 and Supplementary Fig. 1d). Therefore, with our experimentally verified values, there was only a slight improvement in hexamer synchrony in the population when PDDA is included in simulations of either partial reactions or the complete oscillator (Supplementary Figs. 9–11). In the course of our simulations; however, we discovered another effect of PDDA that is even more significant in terms of the resilience of the oscillation under in vivo conditions. In particular, we found an improvement in allowable [KaiA dimer]:[KaiC hexamer] stoichiometry. As the concentration of KaiA is changed relative to that of KaiC (either via random fluctuations or changes in the steady-state concentration), PDDA allows rhythmicity over a broader range of [KaiA dimer]:[KaiC hexamer] ratios (Fig. 6a). This happens because PDDA facilitates the transition of KaiC to the state that binds KaiB, thereby potentiating the formation of the stable KaiA•B•C complex that inactivates KaiA. Therefore, excess KaiA is sequestered and inactivated better in the presence of PDDA. Additionally, within a range of [KaiA dimer]:[KaiC hexamer] ratios that support rhythmicity for both +PDDA and −PDDA (approximately 0.3–2.6),+PDDA maintains an accurate period because the transition of KaiC to the KaiB-binding state is facilitated, so the period remains closer to the typical value of the in vitro reaction (~22 h) than in the case of -PDDA (Fig. 6b, c and Supplementary Fig. 12). A limitation of the current model is a general tendency for increasing oscillatory periods near the non-oscillatory regime for excess [KaiA dimer]:[KaiC hexamer] ratios. While phosphorylation proceeds more rapidly with excess KaiA, additional time is required for sequestration with higher KaiA concentrations. The model indicates that +PDDA can partially mitigate this effect by initiating the dephosphorylation phase earlier.

**Fig. 7:**
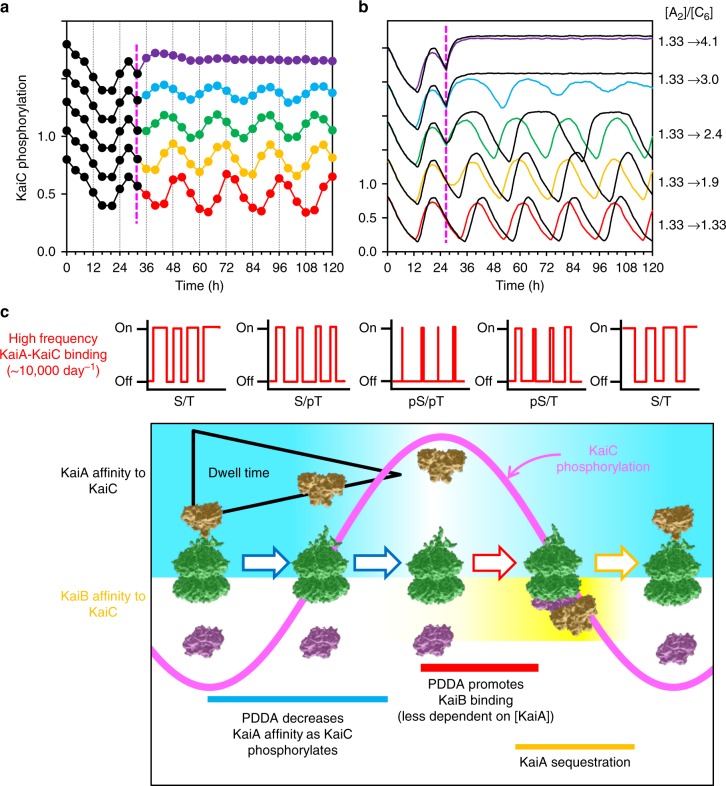
In silico and in vitro tests of PDDA enhancement of resilience. **a** Experimental confirmation of the simulation’s prediction of the effects of an acute KaiA concentration increase during the in vitro oscillation. KaiA concentrations were augmented at hour 32 by the addition of a concentrated KaiA solution to a KaiABC cycling reaction. The traces are vertically offset for clarity. The ordinal scale for each trace is the same as for the control reaction (bottom trace, black/red). **b** Simulation of predictions for the experimental test in **a**, where the ratio of KaiA dimer to KaiC hexamer ([A_2_]:[C_6_]) was increased from 1.33 to 1.9, 2.4, 3.0 or 4.1 at hour 27. Depicted are KaiC phosphorylation patterns resulting from KaiA steps during the dephosphorylation phase with PDDA (+PDDA, colored traces) as compared to the absence of PDDA (−PDDA, black traces). The [A_2_]/[C_6_] values at the far right apply to the experimental data (**a**) and the simulations (**b**). The traces are vertically offset for clarity as in **a**. **c** Model of PDDA’s action. During the phosphorylation phase of KaiC (S/T→S/pT→pS/pT), PDDA causes KaiA affinity for KaiC to decrease progressively, resulting in a shorter dwell time. Therefore, KaiC phosphostatus feeds back upon KaiA/KaiC interaction, changing the pattern of high-frequency KaiA/KaiC binding events and slowing its own rate of phosphoryation. This affects the *k*_D_ of KaiA-stimulated KaiC phosphorylation, thereby influencing the period of the oscillation during the phosphorylation half-cycle. Moreover, PDDA facilitates the pS/pT→pS/T transition (by reducing the pS/T→pS/pT back reaction), enhancing KaiB association and the entry into the dephosphorylation half-cycle. This makes the overall oscillating reaction less sensitive to KaiA:KaiC stoichiometry and thus more resilient to fluctuations of the concentrations of the Kai proteins. KaiC, green; KaiA, gold; KaiB, purple

We explored these predictions of PDDA-enhanced resilience by further numerical simulations and by in vitro experiments. In simulations in which [KaiA dimer]:[KaiC hexamer] was altered by the addition of KaiA to various final concentrations, +PDDA was able to tolerate a larger range of [KaiA dimer]-to-[KaiC hexamer] ratio in terms of amplitude, waveform, and period. When [KaiA] was suddenly increased in simulations by the addition of KaiA to the reaction, the in vitro oscillation continued in the +PDDA system, but rapidly damped with constant KaiA–KaiC affinity (i.e., see ‘−PDDA’ in Fig. 7b). To confirm that the +PDDA simulations can qualitatively predict non-trivial experimental dynamics under KaiA concentration changes, we performed an in vitro experiment where stepwise KaiA concentrations were added to the oscillating system during the dephosphorylation phase as in the simulations (Fig. 7a). Remarkably, we observed that the oscillation indeed continued as predicted from the +PDDA simulation (compare Fig. 7a with Fig. 7b). In particular, while the +PDDA step simulations predict many of the resultant waveform features observed in the experimental data, the non-PDDA simulations predict both waveform and period changes that scale with the magnitude of the KaiA step that were not observed in the experimental data. To be specific, non-PDDA predicts (i) a large period lengthening for the step change of [KaiA_2_]/[KaiC_6_] = 1.33 → 2.4, and (ii) a loss of rhythmicity for the 1.33 → 3.0 step. On the other hand, +PDDA accurately predicts the minor period and amplitude effects in the subsequent rhythmicity that were observed experimentally (Fig. 7a, b).

## Discussion

Single-molecule assessments of molecular activity in real-time allow unparalleled insights into the dynamics of proteins in populations. Coupling HS-AFM with stochastic modeling, this investigation revealed insights into the interactions of circadian clock proteins that promote oscillatory resilience. We report here that the binding of KaiA to the KaiCII tentacles as well as the duration of the bound state is a function of KaiC phosphostatus. As shown in Fig. 7c, PDDA progressively decreases the affinity of the rapid on/off binding of KaiA for the KaiC tentacles as KaiC hexamers become progressively more phosphorylated. The simple and intuitive assumption that longer KaiA-binding durations lead to an enhanced probability of KaiC phosphorylation thereby explains why the rate of KaiA-stimulated KaiC autokinase activity is greater for less highly phosphorylated KaiC hexamers than for more highly phosphorylated hexamers^21^. Therefore, PDDA is a feedback mechanism whereby KaiC’s phosphostatus progressively modulates the kinetics of KaiA binding, allowing the integration of high-frequency interactions into longer term timekeeping. Moreover, PDDA assists this biochemical KaiABC oscillator to keep accurate timing in environments of fluctuating clock protein levels resulting from molecular noise.

Our measurements of PDDA and modeling vindicates the prediction of van Zon and coworkers that PDDA of KaiA for KaiC exists, but we find that the mechanistic significance of PDDA is more far-reaching than that suggested by the model-only approach of van Zon and co-authors^22^. Their model predicted that synchronization can be achieved in the phosphorylation half-cycle of the KaiABC oscillator by differential KaiA affinity (PDDA), and this phenomenon allows sustained oscillations (Supplementary Fig. 1). However, the specific values for PDDA that are necessary for the magnitude of effects they report appear to require that the average KaiA–KaiC affinity parameters change by ~1000 × as a function of KaiC phospho-status (see Supplementary Table 2 of van Zon et al., 2007 which indicates a variation in the KaiA off-rate of 3^6^ = 729). Our empirical measurements of PDDA by HS-AFM range over approximately 50 × at best for KaiC^WT^ (Figs. 4, 5). Within the ~50 × range, the effects of PDDA on inter-hexameric synchronization predicted by van Zon and coworkers in their allosteric ODE model appear to be minor, based on simulations that include site-dependent phospho-transitions and KaiA sequestration.

However, using a stochastic model incorporating KaiC phosphosite-dependent KaiA sequestration (see Supplementary Methods), we find that PDDA values based on our experimental results make the remarkable KaiABC in vitro oscillator resistant to intrinsic noise that can arise in vivo from transcriptional noise, fluctuating metabolites, cell division, etc.^2,3^. PDDA allows a broader range of [KaiA_2_]-to-[KaiC_6_] stoichiometries as permissive conditions for robust rhythmicities (Fig. 6 and Supplementary Fig. 12). As mentioned above in Results, this phenomenon happens because PDDA facilitates the transition of free KaiC to the stable KaiA•B•C complex that sequesters and inactivates excess KaiA. Therefore, as KaiA or KaiC concentrations in the cell fluctuate due to intrinsic noise from transcriptional/translational/degradation or to environmental effects on metabolism/redox, PDDA enhances resilience by making the system less sensitive to excess KaiA. Moreover, introducing PDDA into the model brings the period of the KaiABC oscillator into a shorter, more realistic period range than that obtained without PDDA (Fig. 6c). Note that our model incorporates switching of KaiC to the KaiB-binding state in both the pS/T and pS/pT forms (Fig. 7c) because we are persuaded by phosphomimic data that both the pS/T and pS/pT forms can bind KaiB (i.e., KaiB binds to KaiC-EE and KaiC-DE)^30,31^, so we used this parameter set because we believe it to be more reflective of physiological conditions in the cells. On the other hand, if the more commonly modeled pS/T-only switch is implemented in our model, then +PDDA can be required for sustained oscillations and more generally enables oscillations of an appropriate period over a broader range of effective KaiB on-rates compared to simulations without PDDA (Supplementary Fig. 13c, d). This phenomenon is potentially important because the recent demonstration of slow fold-switching of KaiB introduces a much slower effective rate for the formation of KaiB•KaiC complexes^10^. PDDA can potentially compensate for the effective slow rate of KaiB•KaiC formation correlated with the slow timescale of KaiB fold-switching by promoting the transition to the dephosphorylation phase (see Supplementary Methods).

Regarding extrinsic and intrinsic noise in vivo^2,3^, PDDA provides a satisfying explanation for the excellent resilience of circadian rhythms in cyanobacterial cells undergoing fluctuations in KaiA:KaiC stoichiometry. For example, we reported robust rhythms of KaiC phosphorylation from cells in the face of dramatic variations in KaiC abundance elicited by exposure to differing light/dark conditions (constant light, light/dark cycles of 12 h light/12 h dark, and light/dark cycles of 2 h light/2 h dark)^4^. Moreover, experimental induction of KaiA specifically uncovered a range of KaiA:KaiC wherein the circadian KaiC phosphoryation oscillation (and the rhythm of gene expression it controls) tolerated significant increases of KaiA abundance^4^. These observations are the in vivo correlate of the simulations and experimental manipulations described here (Figs. 6, 7). Generally, PDDA aids nonlinearity in the KaiA-sequestration oscillatory system by pushing the system during the beginning of the phosphorylation half-cycle and releasing (inactivating KaiA by sequestration) at the end of the half-cycle when the phosphorylation peak is reached. This effect is analogous to the variation in the speed of a pendulum as it transitions from maximum speed at the nadir of the path to zero speed at the turning point (maximum excursion).

How do transient, rapid interactions (~1 s) between KaiA and KaiC confer resilience to a 24-h oscillation? The phosphorylation status of the central KaiC clock protein is the key for the KaiABC system, but note that phosphorylation is a general property of clock proteins in all organisms and resilience is crucial for all circadian oscillators. KaiA promotes the autophosphorylation of KaiC, but on average many KaiA-binding/unbinding events are required before a phosphorylation reaction on a T432 or S431 residue occurs. Each KaiA-binding/unbinding event enhances the probability that a phosphorylation reaction occurs, and the basis of our analysis is that the duration of the KaiA binding influences this probability; a longer duration of KaiA binding is more likely to stimulate a phosphorylation event. Our empirical discovery of the PDDA phenomenon adds a critical insight; every phosphorylation event on a KaiC protomer decreases the affinity of KaiA for KaiC (i.e., decreases the duration of KaiA binding to the KaiC tentacles) for subsequent interactions, thereby reducing the probability of KaiA-enhanced phosphorylation (Fig. 7c). Consequently, while each KaiA binding to a KaiC tentacle is a sub-second event, this interaction is ratcheted over the duration of the 12-h phosphorylation half-cycle by KaiC phospho-status. Therefore, each phosphorylation event during the phosphorylation half-cycle calibrates the duration of subsequent KaiA-to-KaiC interactions.

What is the mechanism by which PDDA is accomplished? In a study of KaiA–KaiC interactions, Kim and coworkers concluded that each KaiA-interacting C-terminal tentacle is directly linked by a β-strand segment to the phosphorylatable S431/T432 region^42^. Kim et al. hypothesized that binding of KaiA to a KaiC tentacle pulls on the β-strand linker, causing conformational changes in the S431/T432 region. There are two phospho-sites in each protomer, and therefore 12 sites within the KaiC hexamer; as KaiC is phosphorylated, extra H-bonds are added in this key region of the KaiCII domain that reduce free energy and stabilize KaiCII conformation^47^. This stabilization of the S431/T432 region may radiate back to the linker segment and C-terminal tentacles, thereby modifying the kinetics of KaiA binding. Consequently, KaiC phospho-status is both a marker of circadian phase and a regulator of KaiC’s myriad activities (e.g., ATPase, autokinase, autophosphatase, phosphotransferase, and nanocomplex formation^12^), including modulating the affinity of KaiA interaction.

Phosphorylation is a general property of circadian clock proteins and resilience is crucial for all circadian oscillators^48,49^. Therefore, the properties of modulation of phosphorylation rates at different phases of the rhythm and integration of high-frequency events that we report here are likely to be found in all circadian oscillators, including the insight that molecular noise in vivo might be buffered by fast kinetic binding events among clock proteins. The KaiABC system provides the best biophysical insights to date on the mechanisms by which the phospho-status of clock proteins can biophysically achieve molecular resilience in the face of intrinsic and extrinsic noise that lead to fluctuating concentrations and/or properties of core clock proteins.

## Methods

### Protein purification

Native (unmutated) and mutant Kai proteins from *S. elongatus* were expressed as N-terminal GST fusions in *Escherichia coli* and purified as described previously^19,31,34^. *E. coli* BL21 (Novagen, EMD Millipore) or DH5α (Thermo Fisher Scientific) cells harboring pGEX-based expression construct^8,19,24,31,34^ were grown in 1 liter of Lysogeny broth (LB) medium containing 100 mg ampicillin or carbenicillin, and recombinant protein was expressed by induction with 100 μM IPTG at room temperature (22–24 °C) for 24 h. The cells were harvested by centrifugation and resuspended in 35 mL of extraction buffer containing 50 mM Tris-HCl, pH 8.0, 150 mM NaCl, 1 mM DTT. For the native and mutated KaiC proteins, 5 mM MgCl_2_ and 1 mM ATP were always present in the buffers throughout the purification process. The cells were homogenized by passing through a French pressure cell (5.5 MPa) four times. The cell homogenate was centrifuged at 20,000×*g* for 90 min, and the resulting supernatant was applied to a glutathione-agarose (Pierce, Thermo Fisher Scientific) affinity chromatography column (~1 mL bed volume packed in a PD-10 column, GE Healthcare Life Sciences). After washing the column with 40–60 mL of the extraction buffer, GSH-agarose beads were resuspended in 2 mL of the extraction buffer, and the GST-fusion protein was digested in the column with GST-human rhinovirus 3C (HRV3C) protease at 4 °C overnight. Tag-free protein was eluted from the column with 8 mL of the extraction buffer. The eluted protein was diluted with 20 mM Tris-HCl, pH 8.0, 1 mM DTT (plus 5 mM MgCl_2_ and 1 mM ATP for KaiCs) to reduce NaCl concentration to 90 mM and applied to an anion exchange column (1 mL Q Sepharose Fast Flow, GE Healthcare Life Sciences). For KaiA and KaiC samples, protein was eluted from the column by a stepwise gradient of NaCl (10 mM increments, from 100 to 450 mM) in buffer containing 20 mM Tris-HCl, pH 8.0, 1 mM DTT (5 mM MgCl_2_ and 1 mM ATP for KaiC) using gravity flow of 1.0 or 1.5 mL per step. Since KaiB protein does not bind to Q Sepharose, flow-through from the anion exchange column was collected, concentrated, applied to a gel filtration column (Sephacryl S-100 HR, GE Healthcare Life Sciences, 1.5 cm × 45 cm) and eluted with 20 mM Tris-HCl, pH 8.0, 300 mM NaCl, 0.5 mM EDTA, 1 mM DTT at a flow rate of ~0.25 mL per min. Elution peaks from the ion-exchange or gel filtration column were determined by protein assay and SDS-PAGE. Peak fractions were combined, concentrated (1–3 g L^−1^) and buffer exchanged into 20 mM Tris-HCl, pH 8.0, 150 mM NaCl, 1 mM DTT (plus 5 mM MgCl_2_ and 1 mM ATP for KaiC) by using an Amicon Ultra centrifugal filter unit (MWCO 30 K or 100 K, EMD Millipore). The proteins were >98% pure as determined by SDS-PAGE analysis using Blue silver colloidal Coomassie detection^50^. Protein concentration was determined by the Bradford protein assay using BSA as standard. The purified proteins were snap-frozen in liquid nitrogen and stored at −80 °C until use.

### HS-AFM imaging

HS-AFM experiments were carried out using a laboratory-built High-Speed-Atomic Force Microscope^38,39^. For tapping-mode HS-AFM imaging, a small cantilever with a resonant frequency of ~0.8 MHz, quality factor of ~2 and spring constant of ~0.1 N m^-1^ was oscillated at near the resonant frequency and changes in the oscillation amplitude caused by probe–surface interaction was detected by an optical deflection method. The cantilever’s free oscillation amplitude was set at 1–2 nm and the set-point amplitude for feedback control was approximately 90% of the free oscillation amplitude to avoid an undesirable disturbance of interactions between Kai proteins. KaiC hexamers were immobilized to either bare mica or chemically functionalized mica with 3-aminopropyltriethoxy silane (AP-mica). The observation buffer used was 20 mM Tris-HCl (pH 8.0) and 5 mM MgCl_2_. In some experiments, 1 or 2 mM ATP and/or 150 mM NaCl were present in the buffer, which is indicated in the figure legends. All AFM experiments were carried out at constant temperature (25–28 °C). The KaiC hexamers attached to the mica surfaces as follows: (i) on bare mica, the C-terminal side of the KaiC hexamer attached to the mica (CI end-up orientation), and (ii) on AP-mica, the N-terminal side attached to the AP-mica (CII end-up orientation, Figs. 1, 2).

AP-mica was prepared by placing a droplet (3 μL) of 0.1% 3-aminopropyltriethoxy silane on freshly cleaved mica. After 5 min incubation, the surface was thoroughly washed with 50 μL of pure water. A sample droplet including KaiC hexamers (in the buffer containing Mg-ATP as described above) was placed on the surface and incubated for 5 min. Then residual proteins were washed off with the observation buffer. After confirming by HS-AFM imaging that the KaiC hexamers were homogeneously immobilized on the substrate, a KaiA solution was added into the observation buffer to the final KaiA concentration indicated in the figure legends. After this addition, the buffer solution was well mixed by several cycles of pipetting to provide a uniform concentration of KaiA. With the exception of Figs. 2c, f and Supplementary Figs. 4 and 8 (that include ATP in the observation buffer), all of the data of Figs. 1–5 were obtained in the absence of ATP to prevent KaiC phosphorylation during the brief assay interval, but the presence vs. absence of 1 mM ATP in the incubation buffer did not alter the dynamic interactions between KaiA and KaiC (compare Fig. 3 with Supplementary Fig 4, and Fig. 5 with Supplementary Fig. 8).

### HS-AFM image analyses

To estimate the binding lifetime (τ_bound_) of KaiA•KaiC complexes, the bound-state dwell lifetime was measured from successive HS-AFM images. In addition, the dwell-time between two consecutive events of binding of KaiA dimers to an identical KaiC hexamer was measured to estimate the lifetime of the KaiA-unbound-state (τ_unbound_). The binding and unbinding of KaiA was judged by eye inspection as bright spot appearance or disappearance on a KaiC hexamer. All analyses were carried out for HS-AFM images recorded more than 5 min after KaiA addition to ensure the observation of dynamic interaction events in a well-mixed steady state.

### Construction of simulated HS-AFM images

For the construction of simulated AFM images of KaiC, we used the crystal structure of KaiC (PDB code; 2GBL)^28^ and applied a simple hard sphere model to the individual atoms in the atomic structure. The image construction was accomplished by calculating the contact points between surface atoms of KaiC and a cone-shaped AFM probe with a radius of 0.5 nm and a cone angle of 10°. The simulated AFM image of KaiC-ΔC was constructed using the crystal structure of KaiC from which the C-terminal tentacles were removed.

### Rhythmic in vitro reactions

As described previously^8,34^, In vitro reactions contain 50 mg L^-1^ (1.52 μM) KaiA, 50 mg L^-1^ (4.27 μM) KaiB and 200 mg L^-1^ (3.43 μM) KaiC unless otherwise specified in reaction buffer containing 20 mM Tris-HCl, pH 8.0, 150 mM NaCl, 5 mM MgCl_2_, 1 mM ATP, 0.5 mM EDTA and incubated at 30 °C in a water bath or a thermal block. At each time point, a portion of the reaction was taken for HS-AFM and SDS-PAGE analysis. SDS-PAGE was used to quantify the phosphorylation states of KaiC. The reaction was stopped by mixing with equal volume of 2× SDS-PAGE sample buffer and stored at −20 °C. Different KaiC phosphorylation forms were separated by SDS-PAGE (10% acrylamide gels, 37.5:1 acrylamide:bis-acrylamide). Gels were stained with SYPRO Ruby (Thermo Fisher Scientific) and scanned with Typhoon (GE Healthcare Life Sciences). Digital images were analyzed by NIH ImageJ.

### Statistical analysis

The time constants of the binding lifetime (τ_bound_) and unbound-state (τ_unbound_) of KaiA to KaC were estimated by fitting the histograms with a single exponential curve. The fitting was performed by using the standard regression analysis function of IgorPro. All error bars and data quantifications are expressed as mean ± standard error of the mean (SEM) unless stated otherwise.

### Data availability

Data supporting the findings of this paper are available from the corresponding authors upon reasonable request.

## Electronic supplementary material

Supplementary Information

Description of Additional Supplementary Files

Supplementary Movie 1

Supplementary Movie 2

Supplementary Movie 3

Supplementary Movie 4

Supplementary Movie 5

Supplementary Movie 6

Supplementary Movie 7

Supplementary Movie 8

Supplementary Movie 9

Supplementary Movie 10

Supplementary Movie 11

Supplementary Movie 12

Supplementary Movie 13

Supplementary Movie 14

Supplementary Movie 15

Supplementary Movie 16

Supplementary Movie 17

Supplementary Movie 18
