# Supplementary material for: Revealing circadian mechanisms of integration and resilience by visualizing clock proteins working in real time
Source: Nat Commun. 2018 Aug 14;9:3245. doi: 10.1038/s41467-018-05438-4 (PMC6092398; doi:10.1038/s41467-018-05438-4)
Supplement: Supplementary file 1 — Supplementary Information [file 41467_2018_5438_MOESM1_ESM.pdf]

Supplementary Information

Revealing circadian mechanisms of integration and resilience by visualizing clock proteins working in real time

Tetsuya Mori, Shogo Sugiyama, Mark Byrne, Carl Hirschie Johnson, Takayuki Uchihashi, Toshio Ando

Supplementary Figures

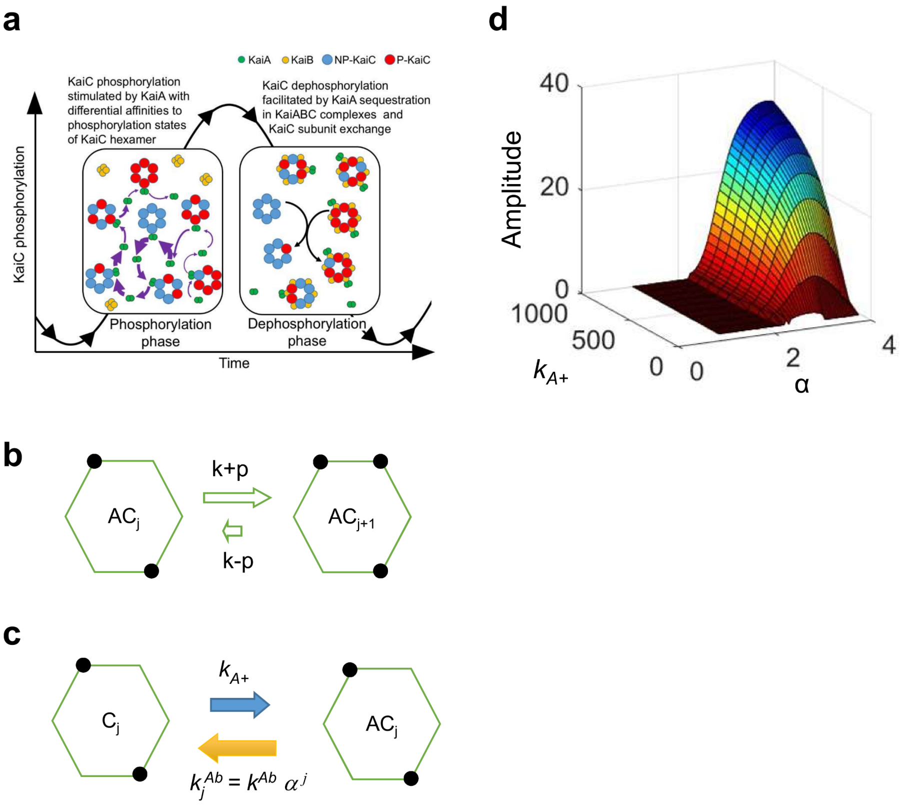

Supplementary Figure 1. Modeling the KaiABC oscillator with Phosphoform-Dependent Differential Affinity (PDDA) of KaiA for KaiC affinity. The original model of van Zon and coworkers[1] suggested differential affinity of KaiA for KaiC during the phosphorylation phase as an explanation for inter-hexamer synchrony in the larger population of hexamers (panel **a**). Their 2007 model posited only two phosphorylation states of KaiC protomers, but we now know that there are at least four different phosphorylation states per monomer and therefore potentially 4^6 states for a KaiC hexamer. Based on their original model, we assumed an idealized hexamer consisting of 6 subunits with 1 phosphorylation site per subunit (panels **b** and **c**). The hexamer has 7 different phosphorylation states (0 to 6 phosphorylations) upon which its binding affinity to KaiA depends (panels **b** and **c**). When KaiA and KaiC are physically associated, the autokinase activity of KaiC is enhanced. The hypothesis predicts that an increased KaiC phospho-status reduces KaiA-KaiC affinity could occur by either decreasing the KaiA-KaiC association rate (k_{on}) or by increasing the dissociation rate (k_{off}) (or both). We explored the model by varying either k_{on} or k_{off} as a function of the KaiC hexamer's phosphorylation status, and confirmed that KaiC-phosphoform-dependent differential affinity (PDDA) of KaiA for KaiC can potentially stabilize oscillations because all KaiC hexamers compete to bind KaiA in the phosphorylation half-cycle, but the KaiC "laggards" (less phosphorylated) are favored in the competition and therefore "catch up" in terms of phosphorylation, and the end result is a more highly synchronized population of KaiC hexamers[1] (panel **d**).

(**a**) Conceptual model of the autonomous synchronization of the KaiABC oscillator by KaiC subunit-exchange, KaiA sequestration, and KaiC phosphorylation state-dependent differential affinity of KaiC for KaiA.

(**b** and **c**) A schematic of the differential affinity of the interaction between KaiA and KaiC (based on van Zon et al, 2007[1]). **b.** Phosphorylation and dephosphorylation of KaiC hexamers in complexes with KaiA; j is a state variable for population phosphorylation (j = 0 to 6 in this model). **c.** Simulating differential affinity of KaiA for KaiC hexamers as a function of KaiC phosphorylation status. The term α is a measure of the affinity of KaiA interaction with KaiC as assessed by the off-rate of KaiA binding; $\alpha = 1$ means there is no difference in KaiA off-rate as a function of KaiC phospho-status, whereas $\alpha > 1$ means that KaiA off-rate speeds up with increasing KaiC phosphorylation as a power law in α.

(**d**) A limited range of α is consistent with sustained oscillations in the model of van Zon & coworkers. The PDDA model is incorporated into a previous model for the KaiC phosphorylation rhythm that includes KaiA sequestration and KaiC subunit-exchange[7]. The relative amplitude of oscillations is shown as α is varied.

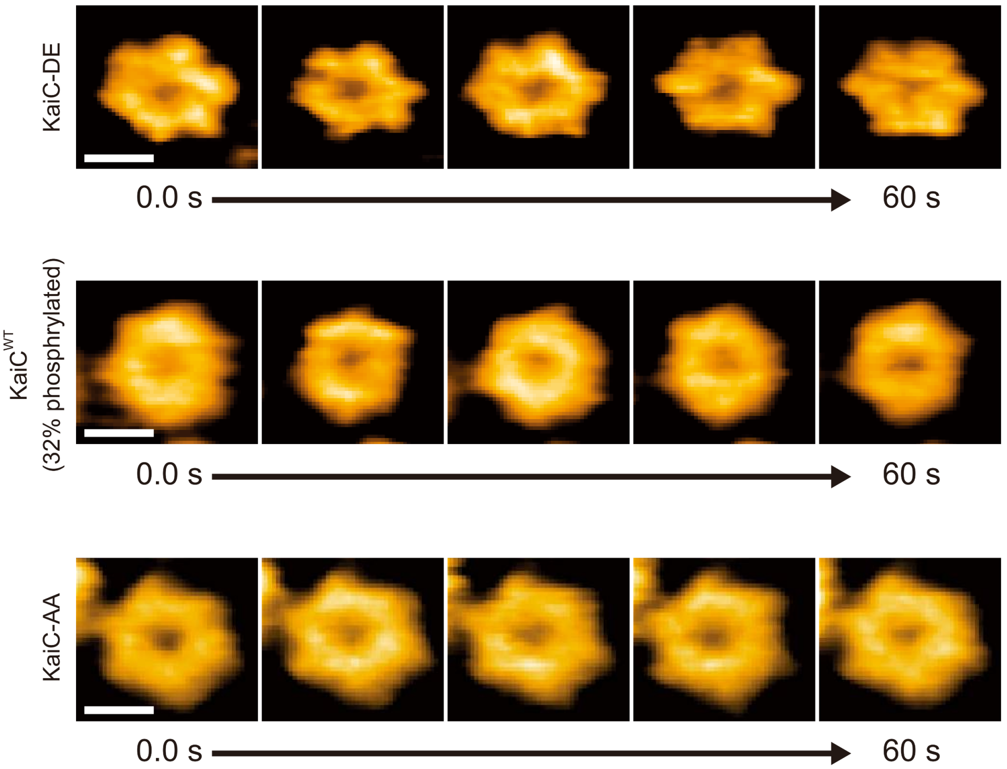

Supplementary Figure 2. Time lapse images of KaiC hexamers attached to bare mica to assess high frequency changes in KaiC conformation. The images show the N-terminal sides of: (upper panel) KaiC-DE (mimic of pS/pT), (middle panel) KaiC^{WT} (32% phosphorylated) and (lower panel) KaiC-AA (mimic of S/T) hexamers in the presence of 2 mM Mg^{2+}-ATP. Imaging was carried out at a frame rate of 1 fps and images every 15 seconds are shown. Scale bars = 5 nm. See also Supplemental Movies 5–12.

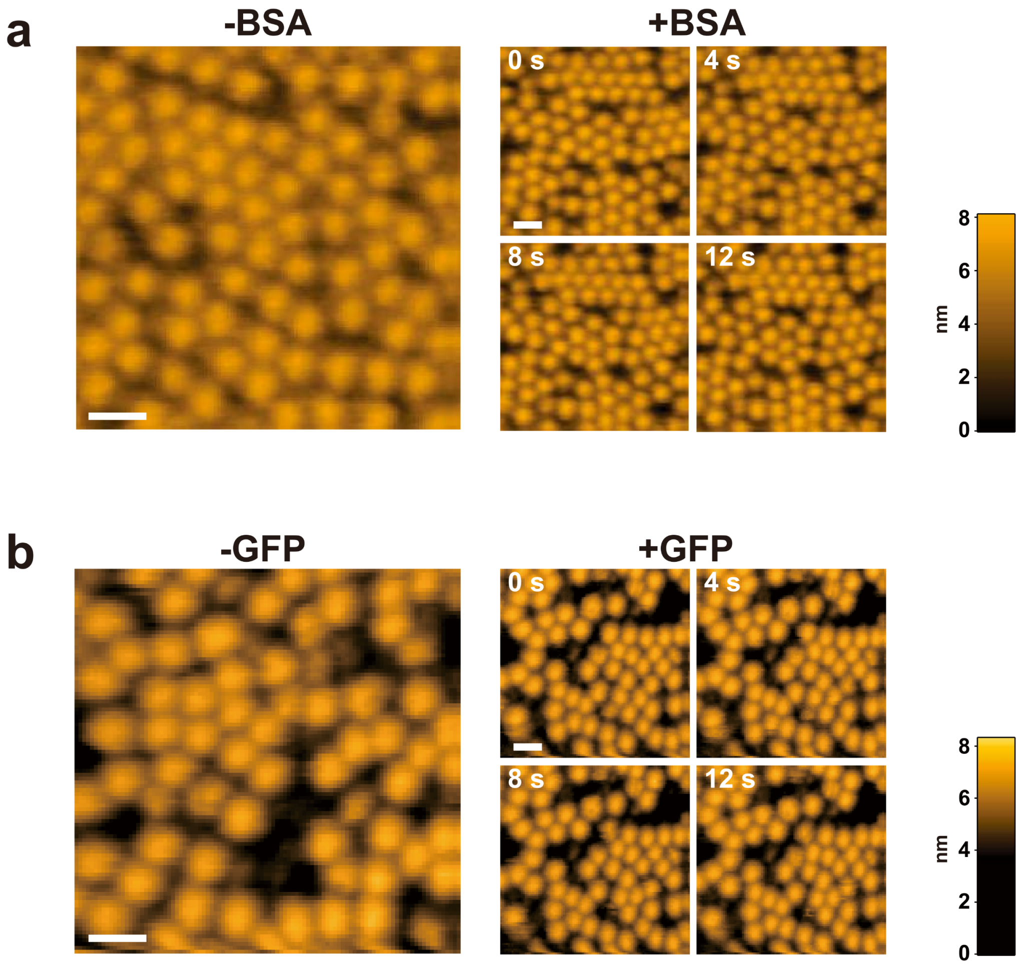

Supplementary Figure 3. HS-AFM observations of KaiCII non-interaction with BSA and GFP (related to Figures 1 & 2). (**a**) Hyperphosphorylated $KaiC^{WT}$ on AP-mica without (left) and with (right) 1 μM BSA in the solution. (**b**) Hyperphosphorylated $KaiC^{WT}$ on AP-mica without (left) and with (right) 1 μM GFP in the solution. All images were acquired at a frame rate of 1.25 fps and the images on the right columns are shown every 4 seconds. Scale bars = 15 nm.

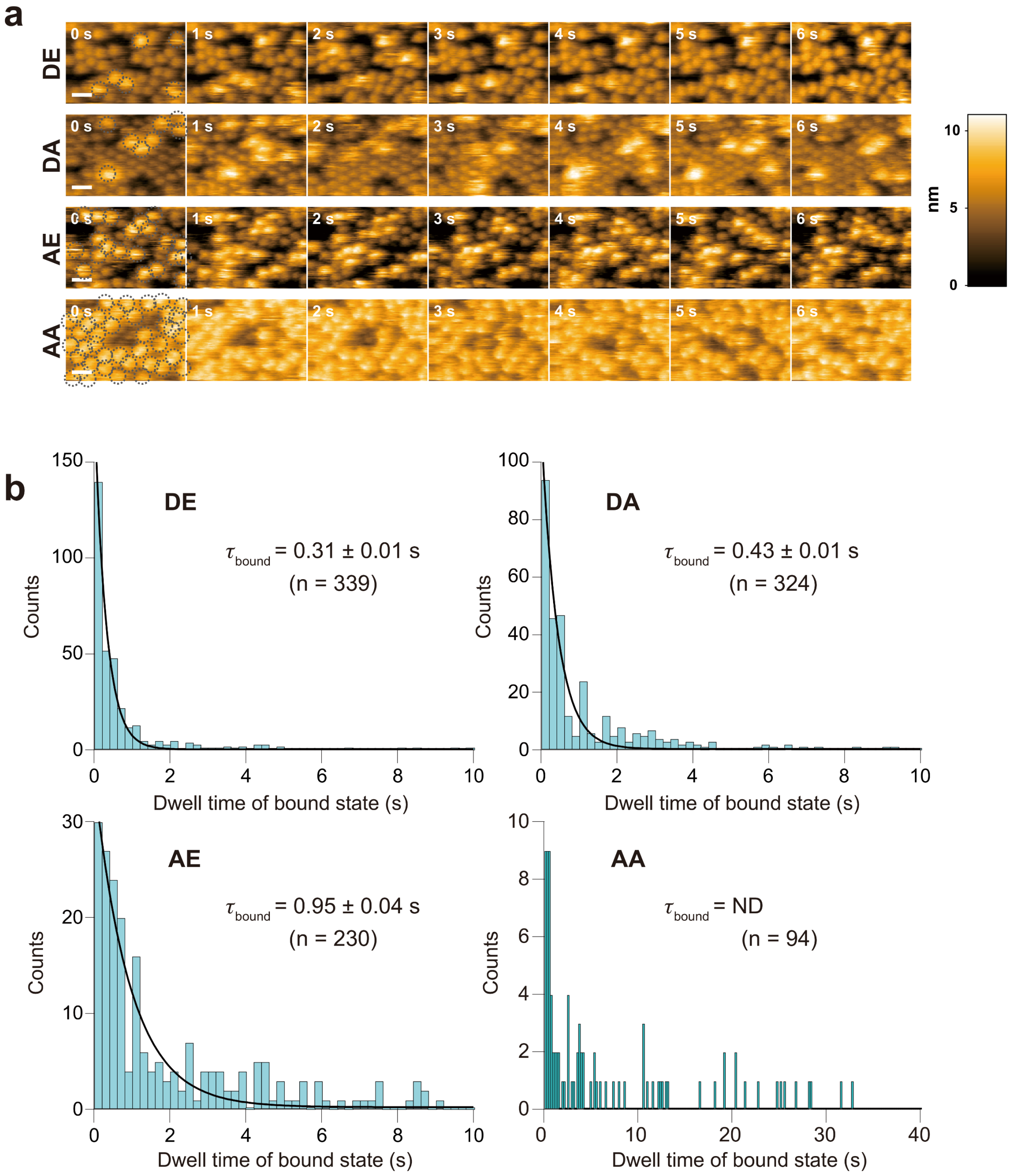

Supplementary Figure 4. Binding affinity of KaiA for KaiC phospho-mimics in the presence of 1 mM ATP (related to Figure 3).

(a) HS-AFM images of various phospho-mimics of KaiC interacting with KaiA. Frame rates, 10 fps. Scale bars = 20 nm.

(**b**) Dwell time analysis for KaiA-bound state of phospho-mimics of KaiC in the presence of 1 mM ATP. The AFM images used in this analysis were captured at 10 fps. Each black solid line overlaid on the corresponding histogram of dwell time was obtained by fitting the histogram to a first order reaction model with a time constant (τ_{bound}) shown, except for the case of AA, where the value of τ_{bound} could not be determined (ND).

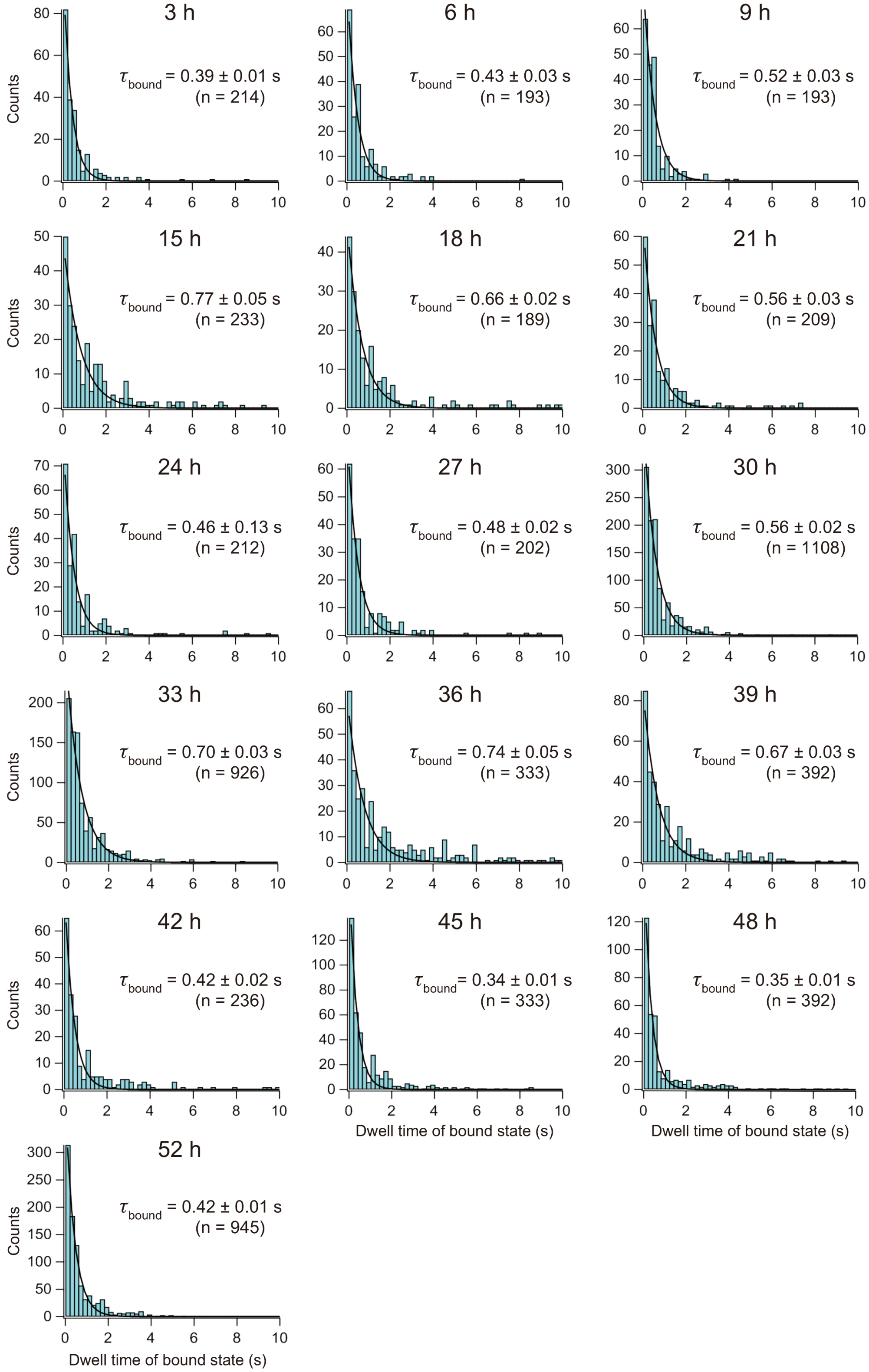

Supplementary Figure 5. KaiA-KaiC binding affinity oscillates with the KaiC phosphorylation rhythm *in vitro* (related to Figure 4). Dwell time analysis of KaiA•KaiC bound state at times from 3 h to 51 h in the *in vitro* KaiABC reaction. Black solid lines are single exponential decay fits yielding the time constant τ_{bound}.

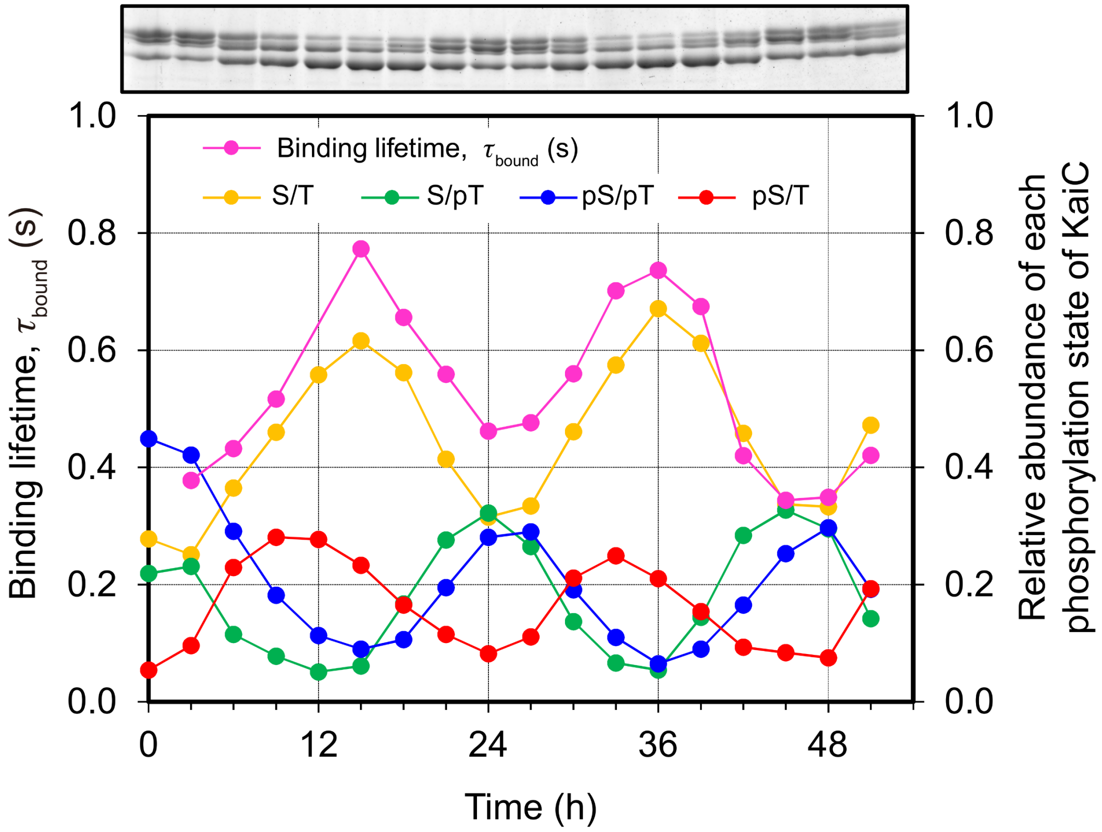

Supplementary Figure 6. Binding lifetime correlates directly with the various phospho-forms of KaiC over the *in vitro* oscillating reaction (related to Figure 4). The relative levels of each phosphoform of $KaiC^{WT}$ (S/T, S/pT, pS/pT, and pS/T) was estimated by SDS-PAGE, and the SDS-PAGE gel image is shown at the top.

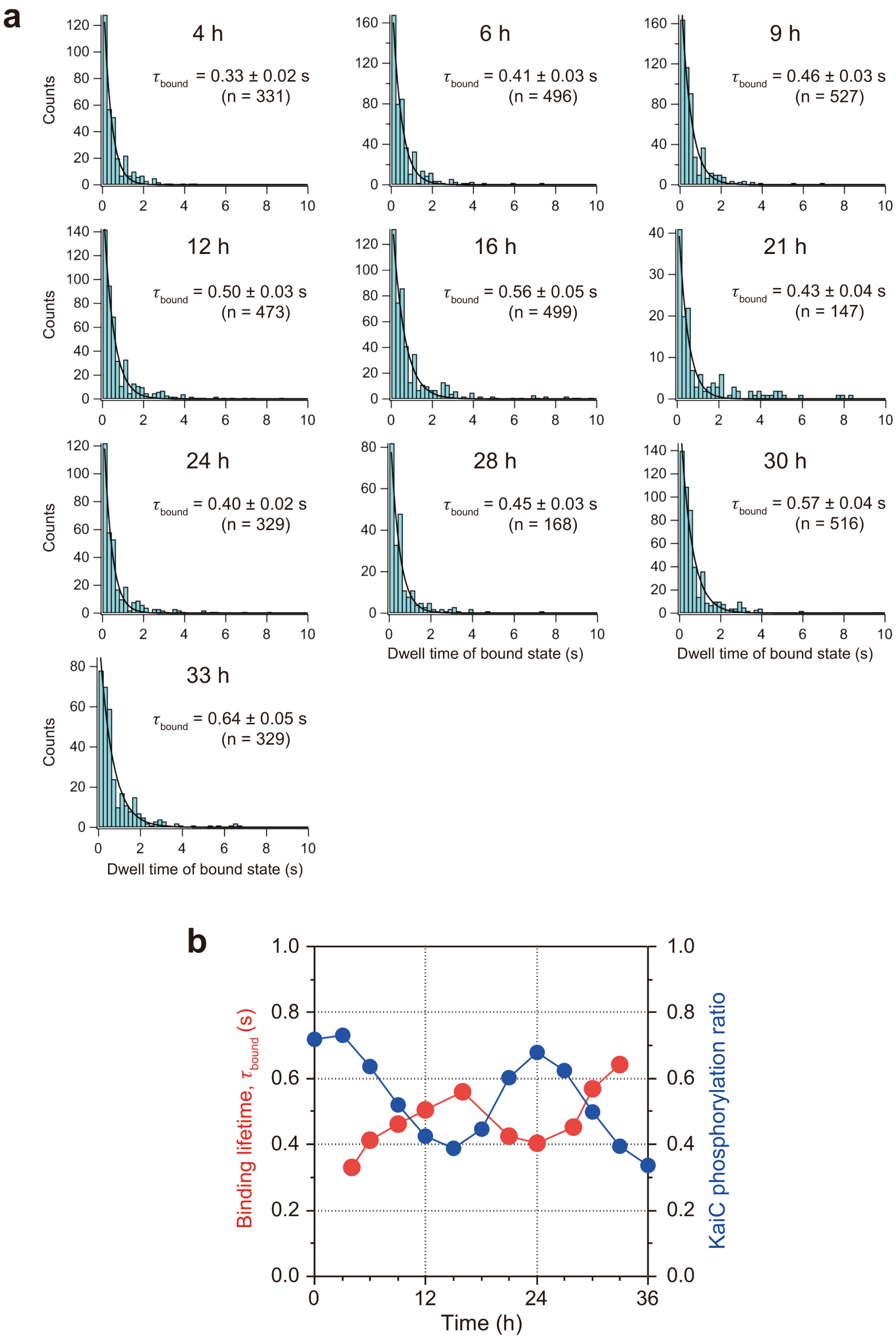

Supplementary Figure 7. KaiA-KaiC binding affinity oscillates with the KaiC phosphorylation rhythm *in vitro* (related to Figure 4). Dwell time analysis of KaiA•KaiC bound state at times from 4 h to 33 h in the *in vitro* KaiABC reaction. The final concentration of KaiA was 1.9 μM. Black solid lines are single exponential decay fits yielding the time constants τ_{bound}. Supplementary Fig. 7 is a replicate experiment to the experiment depicted in Fig. 4.

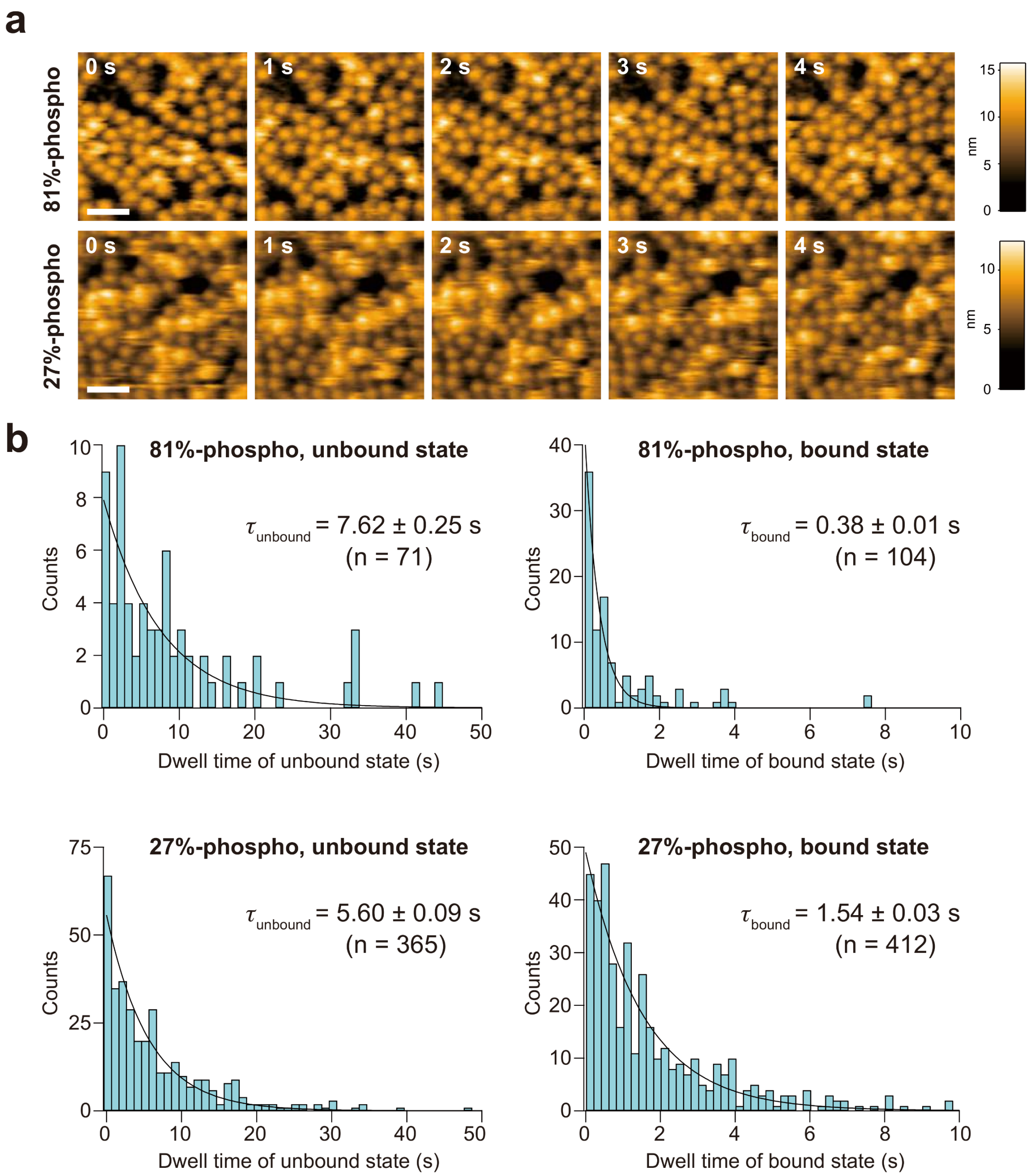

Supplementary Figure 8. KaiA and KaiC interaction in the presence of 1 mM ATP. This figure shows the Phosphoform-Dependent Differential Affinity (PDDA) for the interaction between KaiA and native KaiC assessed as both k_{off} and k_{on} rates by the same methodology shown in Figure 5 except that 1 mM ATP was included in the buffers.

(**a**) HS-AFM images of $KaiC^{WT}$ with 81% (upper panels) or 27% (lower panels) phosphorylated state interacting with KaiA in the presence of 1 mM ATP. Images were acquired at a frame rate of 1 fps. Scale bars = 30 nm.

(**b**) Dwell time analysis of $KaiC^{WT}$ and 0.4 μM KaiA interaction for AFM images acquired at a frame rate of 10 fps. Single-exponential decay fits yield time constants $\tau_{unbound}$ or τ_{bound}.

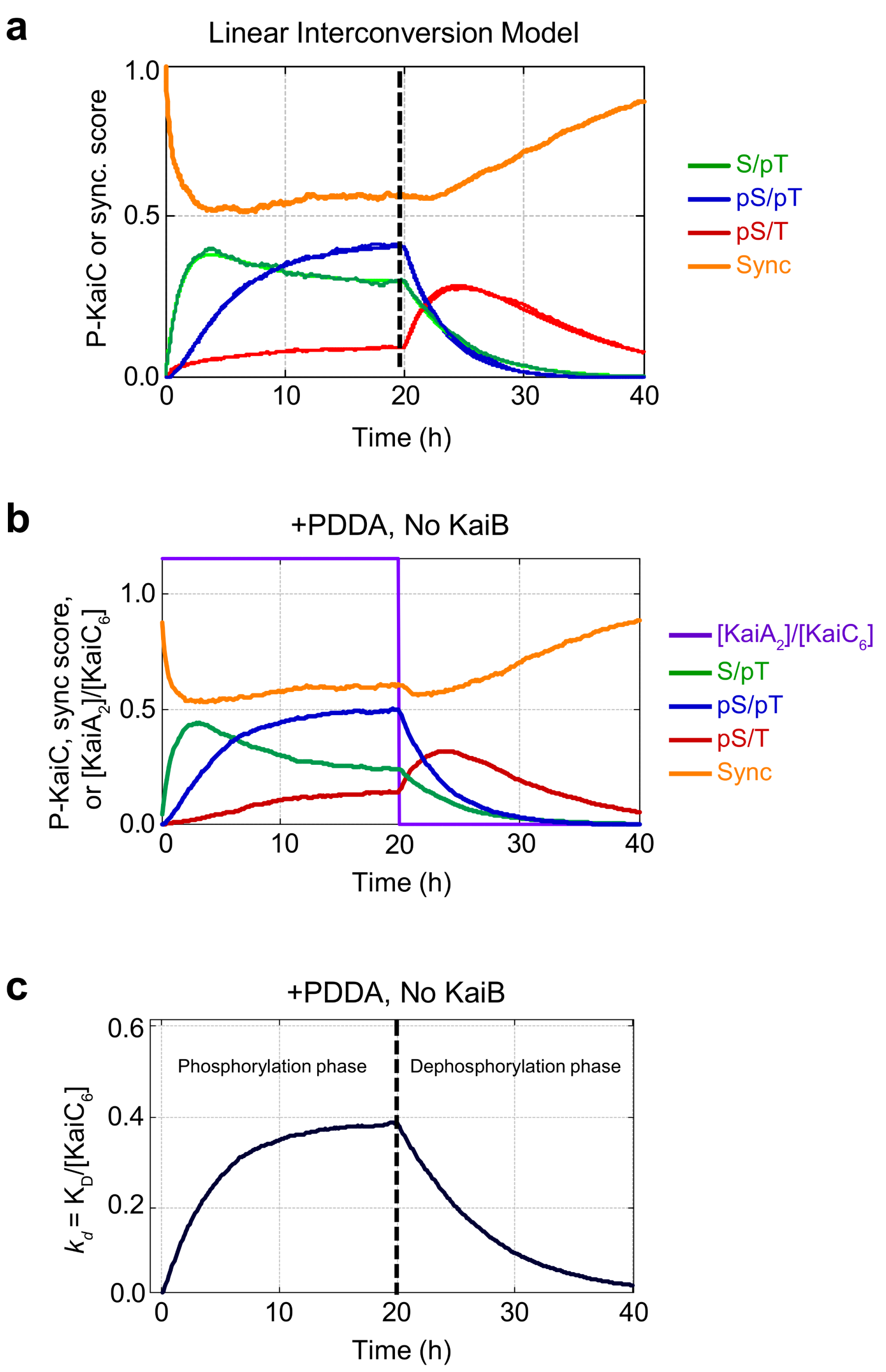

Supplementary Figure 9. Model Simulations of Partial Reactions including PDDA.

(a) Stochastic and deterministic time course simulations of the phosphorylation and dephosphorylation phases assuming linear interconversion between phosphoform states. A measure of hexamer population synchronization is also shown (“Sync”), where higher values indicate better synchronization.

(b) Simulated phosphoform time courses for the phosphorylation phase (KaiA+KaiC) including PDDA and dephosphorylation phase (KaiC alone).

(c) Simulated population average dimensionless KaiA-KaiC dissociation constant using the model for PDDA described in “Computational Methods: Model and Simulations**”** based on the variation in phosphoforms shown in panel b.

a

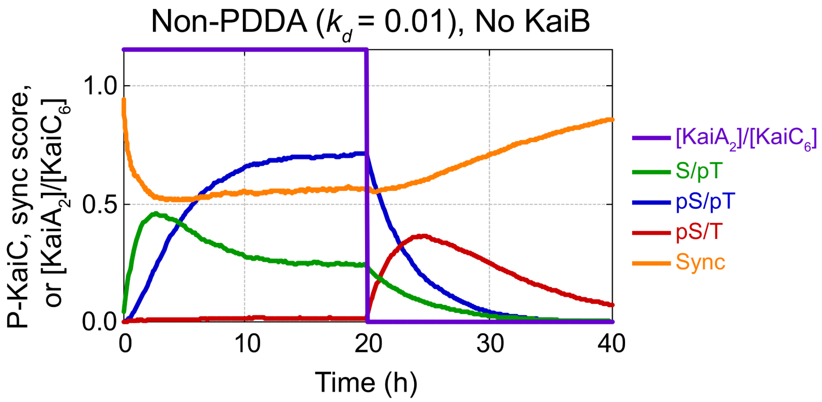

b

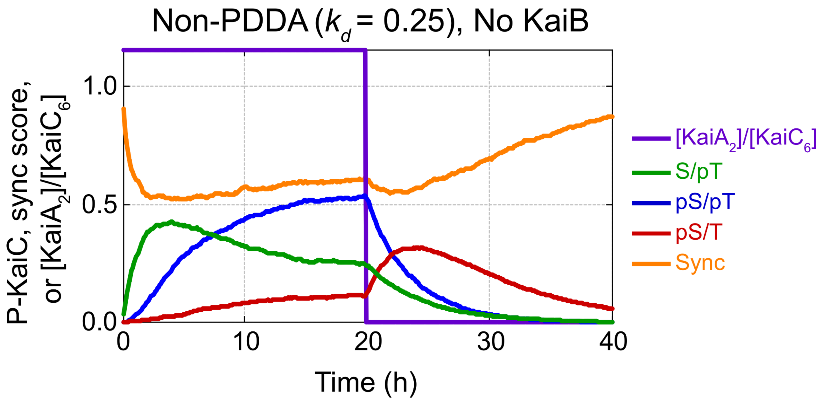

Supplementary Figure 10. **Simulations of Partial Reactions without PDDA**

(a) Sample simulated non-PDDA "control" assuming a locked "high affinity" (k_d = 0.01) for the formation of KaiA-KaiC complexes.

(b) Sample simulated non-PDDA "control" assuming a locked "intermediate" (k_d = 0.25) affinity.

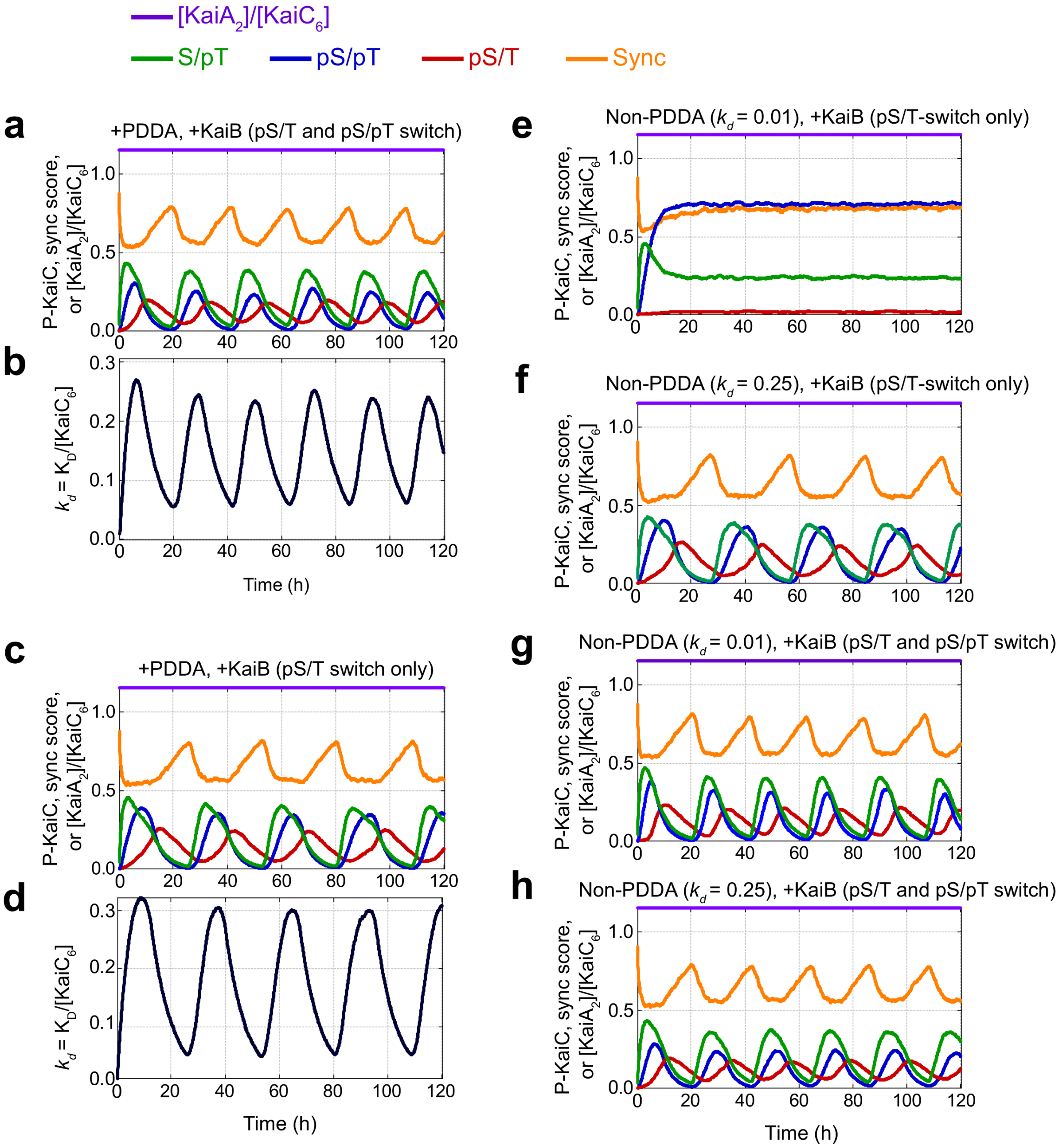

Supplementary Figure 11. **Comparison of Model A and Model B switching +/- PDDA.**

Panels A-H. Sample time course simulations of the KaiA+KaiB+KaiC reaction with phosphoforms and synchronization shown for both Model A (allosteric "pS/T-switch," panels **c**, **e**, **f**) and Model B (dual "pS/T-pS/pT" allosteric switch, panels a, g and h) of KaiB-KaiC complex formation. For +PDDA simulations, the variation in dimensionless KaiA-KaiC dissociation constant is shown (panels **b** and **d**). For non-PDDA "controls" sample simulations with locked "high" affinity (panel1**e** and **g**) and "intermediate" affinity (panels **f** and **h**) are shown. Color of the lines are the same as in Supplementary Figures 9 & 10.

(a ,b) with PDDA (+PDDA), Model B.

(c, d) with PDDA (+PDDA), Model A.

(e) without PDDA, Model A, k_d = 0.01.

(f) without PDDA, Model A, k_d = 0.25.

(g) without PDDA, Model B, k_d = 0.01.

(h) without PDDA, Model B, k_d = 0.25.

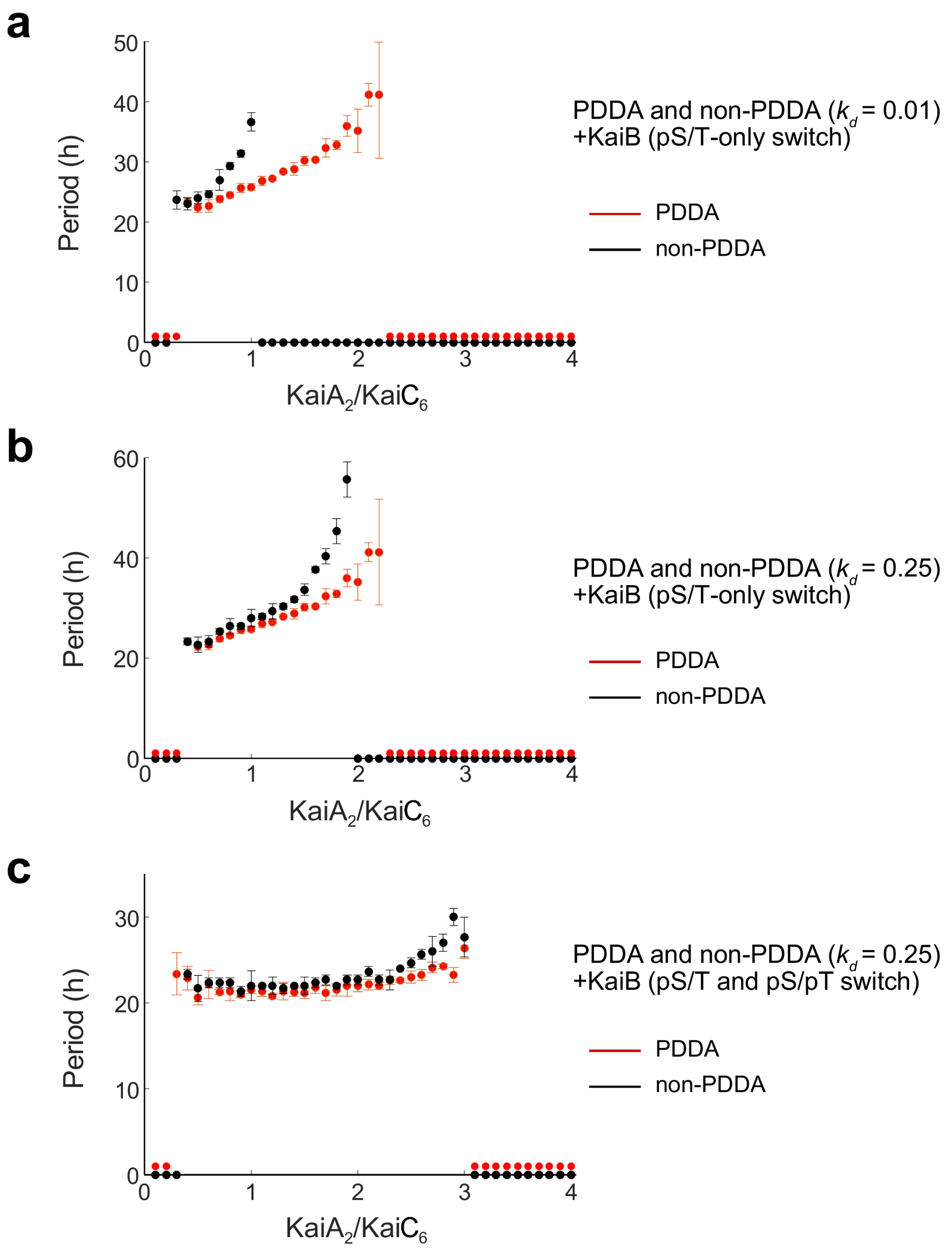

Supplementary Figure 12. Range of allowed oscillatory regimes under Model A vs. Model B.

(a and b) Approximate oscillatory period (and non-oscillatory regime) as KaiA is varied for +PDDA vs non-PDDA using Model A (the pS/T-switch model in locked "high" affinity (k_d = 0.01, panel a) and locked "intermediate" affinity simulations (k_d = 0.25, panel b).

(c) Approximate oscillatory period (and non-oscillatory regime) as KaiA is varied for +PDDA vs non-PDDA using Model B (dual pS/T-pS/pT switch) vs. Model A (locked "intermediate" affinity) Error bars (± S.D.) are from at least 3 replicates with different initial random seeds. Absence of oscillations is indicated as period = 0.

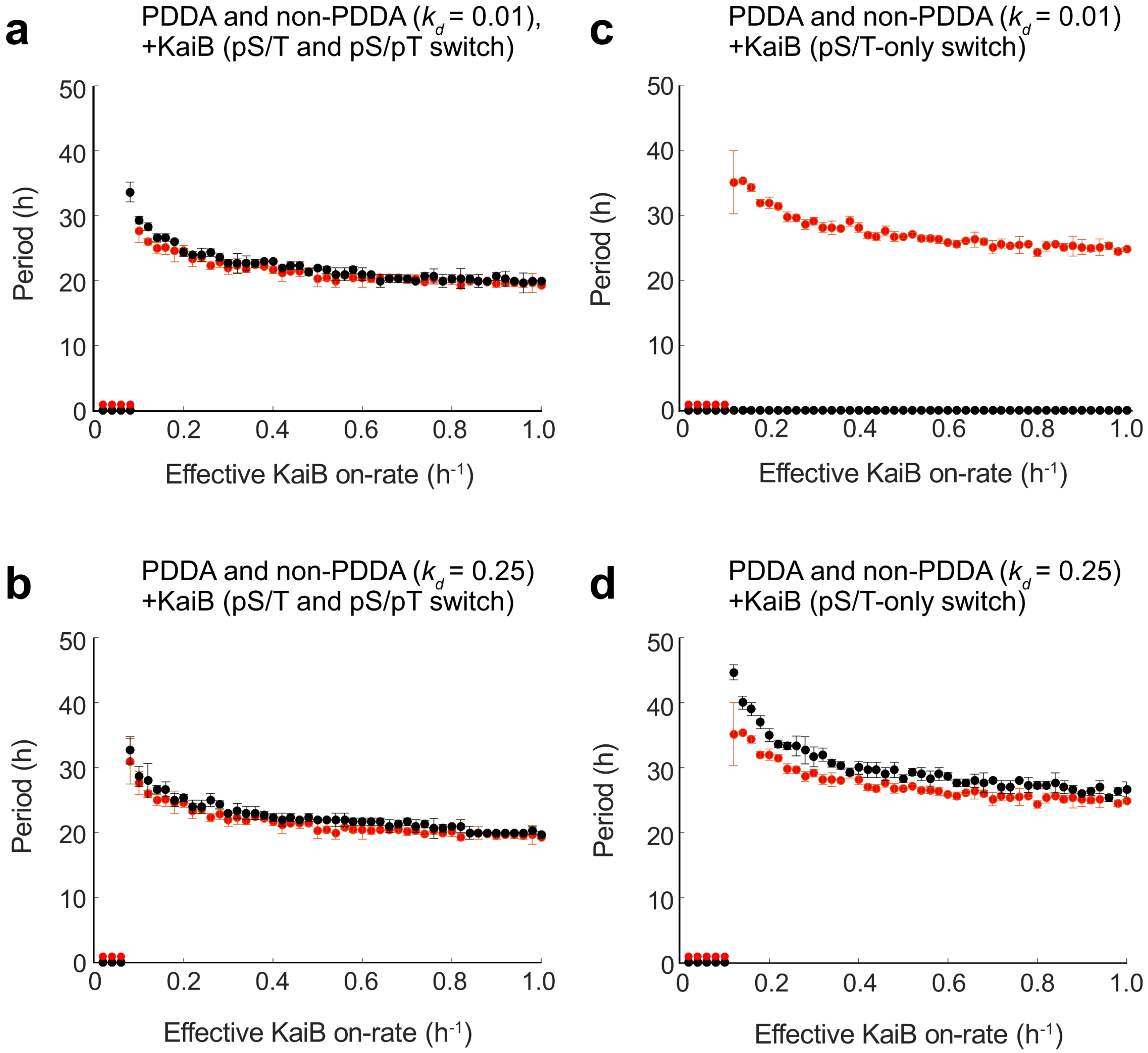

Supplementary Figure 13. Comparison of Model A vs. Model B for rate of KaiB binding. Approximate oscillatory period (and non-oscillatory regime) as the effective KaiB-KaiC association rate is varied for +PDDA vs non-PDDA using Model A (pS/T-switch model, panels **c** and **d**) or Model B (dual pS/T-pS/pT allosteric switch, panels **a** and **b**). Panels **a** & **c** assume locked "high" affinity (k_d = 0.01 for KaiA dissociation), while panels **b** & **d** assume a locked "intermediate" affinity non-PDDA (k_d = 0.25 for KaiA dissociation). Red traces are +PDDA, black traces are non-PDDA. Error bars are ± S.D.

Supplementary Methods

Computational Methods: Model and Simulations

1. Simulating PDDA in a simple phenomenological model (van Zon *et al.* 2007) without site-dependence.
We simulated the simple phenomenological PDDA model of van Zon and coworkers[1] (Equations (3)-(5) in their main text; Equations (23)-(26) in their SI), using a 4th order Runge Kutta ODE solver. The ODEs were all rescaled to the initial KaiC concentration ($[C_0]$) and circadian timescale phosphorylation (k_{ps}), dephosphorylation (k_{dps}), and transition rates (b_0, f_6) of 1.0 hr^{-1} were assumed for simplicity. We investigated the system dynamics as both k_{Af} and k_j^{Ab} were varied, where the latter rate, $k_j^{Ab} = k^{Ab}\alpha^j$ with α > 1 (and j = 0 to 6) was varied in simulations by varying α (Supplementary Fig. 1d). A minimum "slow" KaiA-KaiC off-rate of $k^{Ab} = 0.1$ hr^{-1} was assumed in Supplementary Fig. 1 and the *scaled* KaiA on-rate was varied, $k_{A+} \equiv k_{Af}[C_0]$ (hr^{-1} unit). The initial conditions in simulations were $[C_0] = 1$ (μM), $[A_0]/[C_0] = 0.25$, with the rest of the complexes initially set to zero. These simple PDDA model simulations suggest an optimal range of PDDA (α), where either insufficient PDDA (low α) or excess PDDA (high α) do not allow stable oscillations; furthermore, the amplitude variation as a function of α shows that the system smoothly transitions through a maximum as α increases monotonically. The latter effect of loss of oscillations for high α can be understood qualitatively from the model; for sufficiently large α the system will be unable to phosphorylate the higher states (j = 4 to 6, for example) as the probability of forming higher phosphorylated KaiA-KaiC complexes becomes too low. We expect this result (a limited range of PDDA which improves oscillatory dynamics) to be a general result in more complicated PDDA models which we investigate below.

2. KaiABC PDDA site-dependent model and numerical simulations

2.1 Simulating site-dependent phosphorylation and de-phosphorylation
To investigate the possible role of PDDA in system dynamics including site-dependence we updated a previously published stochastic matrix model (a matrix of *N* rows = *N* hexamers by 6 columns = protomers) to simulate KaiC hexamer dynamics and phosphoform transitions using Monte Carlo for reaction probabilities (per timestep and per molecule). Each monomer can be in one of four phosphostates (*U= S/T, T = S/pT, D = pS/pT, S = pS/T*) encoded by the labels (0,1,2,3).

Previously published linear interconversion rates (fitted to partial reaction data in the presence or absence of KaiA) between the four phosphoform states (*S/T → S/pT*, etc) were applied as transition probabilites (per time) to single protomers in simulations and compared with a 4th order Runge-Kutta solution to the corresponding linear differential equations to confirm the stochastic simulation algorithm (Supplementary Fig. 9a). Previously published linear interconversion rates between phosphoforms were initially tested[2], Supplementary Table 1.

We define a measure of the degree of synchronization in the hexamer population by counting the number of each phosphoform type in each hexamer and computing the Euclidean "distance" between hexamer pairs, $D_{i,j} \equiv \sqrt{\sum_{\beta=1}^{4} \left[N_i(\beta) - N_j(\beta)\right]^2}$ where $N_i(\beta)$ refers to the number of each phosphoform type, $\beta = \{S/T, pS/T, etc\}$ in the *i*-th hexamer. This distance is then summed over all hexamer pairs in the population; lower values indicate greater hexamer-hexamer synchronization (a completely dephosphorylated, and hence perfectly synchronized population, has $\sum D_{i,j} = 0$). Simulations yield scores in the range $0 < (1/N_{hex}^2)\sum D_{i,j} < 5$ so a convenient normalization and rescaling is to define

the population synchronization index, $S = 1 - (0.2/N_{hex}^2)\sum D_{i,j}$. In the linear interconversion simulations (without PDDA) of the phosphorylation and dephosphorylation phases synchronization of the population decreases during the phosphorylation phase and gradually increases again during dephosphorylation (Supplementary Fig. 9a). In all stochastic simulations we also allowed for the possibility of "monomer exchange" (random shuffling of monomers among hexamers) between any hexamers during any phase[3–5]; the default rate of exchange in simulations was set faster than the fastest transition rate; for the simulations shown in the manuscript a monomer exchange rate of 1.0 hr^{-1} was used unless stated otherwise.

Supplementary Table 1: Linear Interconversion Rates (from Rust *et al.* 2007[2])

KaiA + KaiC ([A] =1.3 μM, [C] = 3.4 μM)	*Forward rate (hr^{-1})*	*Backward rate (hr^{-1})*
S/T ↔ S/pT	0.36	0.27
S/T ↔ pS/T	0.04	0.01
S/pT ↔ pS/pT	0.16	0.13
pS/T ↔ pS/pT	0.38	0.07
KaiC alone ([C] = 3.4 μM)	*Forward rate (hr^{-1})*	*Backward rate (hr^{-1})*
S/T ↔ S/pT	0.00	0.21
S/T ↔ pS/T	0.00	0.11
S/pT ↔ pS/pT	0.00	0.00
pS/T ↔ pS/pT	0.00	0.31

2.2 Simulating KaiA-KaiC reactions and PDDA

Previous fits to partial reaction data parameterized the effect of varying KaiA concentration on the phosphoform transition rates whereas we wish to directly investigate the effect of PDDA of KaiA interacting with KaiC. We assume that these previously estimated effective phospho-transition rates (with rates fitted to the linear model above) are physically a result of (differential) on-off kinetics of KaiA to KaiC as suggested by the experimental data.

KaiA on-off dynamics occur much faster (timescale s^{-1}) than the effective phosphoform transition rates (timescale hr^{-1}) so that simulating KaiA-KaiC reactions in "pseudo" steady state should be a reasonable approximation for estimating transient KaiA-KaiC complex formation in Monte Carlo simulations. Assuming a 1:1 binding model (for free KaiA dimer to KaiC hexamer) with on-rate k_+ and off-rate k_- we compute the steady-state (expected value) fraction of KaiA-KaiC complexes that form given some initial concentration of *free* (non-sequestered) KaiA. Let $x = [AC]/[C_0]$ and $b = 1 + a_0 + k_d$ where $k_d = k_-/(k_+[C_0])$ is a (scaled) dimensionless dissociation constant, $[C_0]$ is the initial concentration of KaiC and $a_0 = [A]_0/[C_0]$ is the initial (fraction) of *free* dimeric KaiA to initial total hexameric KaiC (fixed). Then the steady-state fraction of complex (x^*) that forms is the physical solution of :

$$x^* = 1/2\left(b \pm \sqrt{(b^2 - 4a_0)}\right)$$

PDDA is simulated by varying the dissociation constant (k_d) for the 1:1 binding model based on hexamer composition. In simulations the steady-state calculation is used to estimate the probability of KaiA-KaiC complex formation based on a hexamer's composition and a PDDA model is a specification of k_d in terms of phosphoform composition. We assume a simple phenomenological model where the

scaled dissociation constant varies linearly in terms of the number of each phosphoform type:

$$k_d = k_{d0} + c\ (\ \gamma_1 N(T) + \gamma_2 N(D) + \gamma_3 N(S)\ )$$

where $N(T)$ is the number of S/pT protomers in the hexamers, etc., and $\gamma_i > 0$ are relative weights for altering the dissociation probability of the KaiA-KaiC complex for each phosphoform type (increased phosphorylation of a hexamer lowers the probability of complex formation), k_{d0} is the dissociation constant for completely unphosphorylated KaiC, and $c > 0$ is a scaling factor parameterizing the overall strength of PDDA ($c = 0$ corresponds to "non-PDDA" in the main manuscript figures). Relative weights were selected based on the measured relative off-rates (τ_{bound}) from the experimental data in the ratio $\gamma_1 : \gamma_2 : \gamma_3 = 1.0 : 3.9 : 2.3$ (Fig. 3). The model assumes the dissociation rate(s) are inversely proportional to the measured τ_{bound} values, so we chose ratios suggested by Fig. 3.

To be consistent with the apparent high affinity measured in the mutant phospho-mimic of the dephosphorylated state (KaiC-AA, Fig. 3), we set the (minimum) dissociation constant of KaiA from unphosphorylated KaiC, $k_{d0} = 0.01$. The weights chosen imply a maximum value of $k_d \sim 20c$ and since previous groups have estimated a population average K_D in the range 1-3 μM [2,3], these measurements together suggest $c < 0.05$ as a constraint. In simulations a range of PDDA of approximately $0.01 < k_d < 1.0$ is thus possible as the phospho-composition of a hexamer varies. For simulations we chose an intermediate value of $c = 0.025$ which, using previously estimated maximal phospho-transition rates, reproduces partial reaction phosphoform dynamics similar to previously published experimental data[2] (Supplementary Fig. 9b); the simulated *population average* affinity varies by approximately a factor of 3 for the standard parameters given above (Supplementary Fig. 11b,d). The partial reaction simulations indicate that the inclusion of PDDA increases the relative fraction of pS/T states at the expense of pS/pT states which would be expected from a decreased affinity of KaiA for more highly phosphorylated hexamers. The transition rates assumed for simulation figures in the manuscript are given below (Supplementary Table 2), with a physical interpretation that these rates reflect probabilities (per time) of the phospho-transitions when bound-state KaiA-KaiC complexes transiently form. Since PDDA results in dephosphorylation transitions the rates are modified from the original published values. We also considered additional non-PDDA controls ($c = 0$, varying k_{d0}) to determine the dynamical effect of PDDA, in which non-PDDA can promote the pS/pT → pS/T transition (Supplementary Figs. 10b, 11f, 11h).

Supplementary Table 2: Assumed maximum phosphoform transition rates (adapted from Rust *et al.* 2007[2])

KaiA-KaiC complexes	*Forward (hr^{-1})*	*Backward (hr^{-1})*
S/T ↔ S/pT	0.50	0.05
S/T ↔ pS/T	0.01	0.05
S/pT ↔ pS/pT	0.30	0.10
pS/T ↔ pS/pT	0.50	0.00
KaiC alone	*Forward (hr^{-1})*	*Backward (hr^{-1})*
S/T ↔ S/pT	0.00	0.20
S/T ↔ pS/T	0.00	0.15
S/pT ↔ pS/pT	0.00	0.00
pS/T ↔ pS/pT	0.00	0.27

2.3 Simulating KaiB-KaiC reactions and KaiA sequestration
PDDA of KaiA for KaiC by itself is insufficient for generating oscillations in population phosphorylation as the KaiA-KaiC partial reactions do not appear to oscillate (experimentally) for any choice of initial KaiA and KaiC concentrations. KaiB seems to be required to produce sustained population dephosphorylation by sequestration of KaiA in stable complexes, thereby removing "active" KaiA. Based on previous experiments and models[2,6–12], we consider in simulations two simple "switch" based models for KaiB binding: (A) a pS/T-based switch and (B) a dual (pS/T and pS/pT) switch. In Model A, a minimum number of pS/T protomers per hexamer is required to initiate KaiB binding to an individual hexamer (Supplementary Fig. 11c-f). In Model B, a minimum number of pS/T or pS/pT protomers per hexamer is required to initiate KaiB binding (Supplementary Fig. 11 a-b, g, h). Model B was used in the simulations in Figures 6 and 7 because we are persuaded that there are effective allosteric switch-like mechanisms for KaiB binding[6,9]. Provided this condition is met, KaiB-KaiC complexes form at some rate, k_{+B}. Each of these KaiB-KaiC complexes is assumed to rapidly sequester, on average, n_{seq} free KaiA[13], so that

$$A_{free} = A_0 - n_{seq}\,(BC) \qquad (A_{free} > 0)$$

where *(BC)* indicates the number of KaiB-bound KaiC. Given a KaiB-KaiC complex we allow a general (slow) off-rate for de-sequestration of KaiA and release of KaiB, $k_{-B.}$ This rate constant needs to be sufficiently slow so that sequestration is effective, yet the inclusion of such a rate allows a "small" fraction of free KaiA during the dephosphorylation phase, which is consistent with the experimental observations of Clodong and co-authors and Kageyama and co-authors[3,14]. When a given hexamer dephosphorylates, we assume the rate of KaiB-KaiC complex dissociation (k_{relax}) occurs on a similar timescale (~1.0 hr^{-1}) to the fastest dephosphorylation reactions – this transition is implemented with a similar simple threshold based on the number of pS/T protomers per hexamer falling at or below some minimum value, $B_{-,thresh}$. We use the following parameters summarized in Supplementary Table 3 to simulate the KaiB-KaiC and KaiA-KaiB-KaiC interactions:

Supplementary Table 3: Parameters for KaiB-KaiC protein-protein interactions

Average number of KaiA sequestered per KaiB-KaiC complex	n_{seq} = 4
<u>Model A</u>: Threshold pS/T protomers required for KaiB-KaiC complex formation	$B_{+,pS/T}$ = 2
<u>Model B</u>: Threshold pS/T or pS/pT protomers required for KaiB-KaiC complex formation	$B_{+,pS/T}$ = 2, $B_{+,pS/T+pS/pT}$ = 3
Threshold pS/T states required for KaiB-KaiC complex dissociation	$B_{-,thresh}$ = 0
KaiB on-rate	k_{+B} (varied in simulations)
General KaiB off-rate	k_{-B} = 0.01 hr^{-1}
KaiB-off rate for $N(pS/T) \leq B_{-,thresh}$	k_{relax} = 1.0 hr^{-1}

Assuming 1:1 binding and scaling all concentrations by the initial (fixed) KaiC hexamer concentration (3.4/6 µM standard) to dimensionless values gives a scaled KaiB on-rate ($k_{+B}[C_0]$, units hr^{-1}) varied in the manuscript figures.

In Model B when PDDA is implemented, the population average (dimensionless) dissociation constant of KaiA from KaiC (k_d) oscillates between about 0.1 and 0.3 (Supplementary Fig. 11, panels b & d).

Therefore, for comparisons with non-PDDA controls we also considered both a locked high affinity constant of KaiA for KaiC, k_d (k_d = 0.01) and an intermediate affinity k_d (k_d = 0.25) for the non-PDDA controls in Supplementary Figs. 10b, 11fh, 12bc, 13bd.

2.4 Simulation of the KaiA-KaiB-KaiC Oscillatory system, Oscillatory parameter space, and Effect of PDDA

A simulation is specified by choosing the number of hexamers (N_{hex} = 1000 hexamers is typically used), the *initial* ratio of KaiA dimer to KaiC hexamer (A_2/C_6), the phosphoform transition rates above, the KaiA-KaiC complex formation parameters, and the KaiB-KaiC parameters listed above (we assumed non-limiting KaiB). A typical simulation output for N = 1000 hexamers, initial A_2/C_6 = 1.15, +/- PDDA indicates the onset of oscillations from KaiA (autocatalytic) sequestration by KaiB-KaiC complexes (k_{+B} = 0.5 hr^{-1} shown) in both models, Supplementary Fig. 11a,c. An additional non-PDDA control (c = 0, k_{d0} = 0.25) was also used to determine the dynamical effect of PDDA, in which we allow non-PDDA to promote the pS/pT → pS/T transition in both models (Supplementary Fig. 11f,h). The general qualitative modeling results were checked for a range of PDDA strengths (parameter c). For varying KaiA concentrations (Supplementary Fig. 12a-c) and varying KaiB effective on-rates (Supplementary Fig. 13a-d), oscillatory period estimates were based on peaks and troughs from smoothed total percent phosphorylation simulation output data for each simulation run; the existence or non-existence of oscillations from such a simulation run was typically based on ~200 h simulated time course intervals. The resulting time course simulations were visually inspected to confirm the absence or presence of noticeable oscillations for Fig. 6 in the main manuscript. Simulations were repeated using varying initial random seeds to check that stochastic effects did not change the general qualitative conclusions from comparison of simulations +/- PDDA.

PDDA enhances resilience whether Model A or Model B switching is considered. As discussed in the main text, PDDA in Model B enhances resilience to noise that is due to fluctuations in KaiA:KaiC stoichiometry. In Model A, without PDDA the transition from the phosphorylation phase to the dephosphorylation phase (pS/pT→pS/T) is disfavored because there is no inactivation of KaiA (by sequestration of KaiA) until pS/T becomes dominant, and until that happens, KaiA continues to stimulate the phosphorylation of KaiC to the pS/pT state. However, with PDDA the transition can be promoted by the reduced affinity of KaiA to hyperphosphorylated (pS/pT) KaiC. The pS/T-only switch (Model A) has been used to simulate the cyanobacterial clock mechanism in oft-cited models (e.g., Rust *et al.* 2007[2]), and in this case +PDDA enables oscillations of an appropriate period over a broader range of KaiB on-rate (e.g., compare Supplementary Fig. 13c/d with Supplementary Fig. 13a/b). Most dramatically, Model A (pS/T-only switch) in the absence of PDDA and with the high affinity (k_d = 0.01) values we measured for the dephosphorylated state (KaiC-AA, Fig. 3) does not oscillate for any effective KaiB on-rate (Supplementary Fig. 13c), assuming the standard concentrations of KaiA and KaiC (1.3 and 3.4 μM, respectively). This phenomenon is potentially important because the demonstration of slow “fold-switching” of KaiB introduces a much slower effective K_{+B} term for KaiB binding to $KaiC^{pS/T}$ than is possible to accommodate in previous models unless an unrealistic “hard” KaiA sequestration is invoked[10]. Conversely, PDDA can generate stable oscillations for a wide range of physiologically reasonable effective KaiB/KaiC association rates, and therefore, whichever model is used for the KaiC phosphostatus that promotes KaiB binding, PDDA plays a crucial role to enhance resilience.

Supplementary References

1. van Zon, J. S., Lubensky, D. K., Altena, P. R. H. & ten Wolde, P. R. An allosteric model of circadian KaiC phosphorylation. *Proc. Natl. Acad. Sci. U. S. A.* **104,** 7420–7425 (2007).
2. Rust, M. J., Markson, J. S., Lane, W. S., Fisher, D. S. & O'Shea, E. K. Ordered phosphorylation governs oscillation of a three-protein circadian clock. *Science* **318,** 809–812 (2007).
3. Kageyama, H. *et al.* Cyanobacterial circadian pacemaker: Kai protein complex dynamics in the KaiC phosphorylation cycle in vitro. *Mol. Cell* **23,** 161–171 (2006).
4. Mori, T. *et al.* Elucidating the ticking of an in vitro circadian clockwork. *PLoS Biol* **5,** e93 (2007).
5. Ito, H. *et al.* Autonomous synchronization of the circadian KaiC phosphorylation rhythm. *Nat. Struct. Mol. Biol.* **14,** 1084–1088 (2007).
6. Nishiwaki, T. *et al.* A sequential program of dual phosphorylation of KaiC as a basis for circadian rhythm in cyanobacteria. *EMBO J.* **26,** 4029–4037 (2007).
7. Qin, X. *et al.* Intermolecular associations determine the dynamics of the circadian KaiABC oscillator. *Proc. Natl. Acad. Sci. U. S. A.* **107,** 14805–14810 (2010).
8. Phong, C., Markson, J. S., Wilhoite, C. M. & Rust, M. J. Robust and tunable circadian rhythms from differentially sensitive catalytic domains. *Proc. Natl. Acad. Sci. U. S. A.* **110,** 1124–1129 (2013).
9. Lin, J., Chew, J., Chockanathan, U. & Rust, M. J. Mixtures of opposing phosphorylations within hexamers precisely time feedback in the cyanobacterial circadian clock. *Proc. Natl. Acad. Sci. U. S. A.* **111,** E3937-3945 (2014).
10. Chang, Y.-G. *et al.* Circadian rhythms. A protein fold switch joins the circadian oscillator to clock output in cyanobacteria. *Science* **349,** 324–328 (2015).
11. Oyama, K., Azai, C., Nakamura, K., Tanaka, S. & Terauchi, K. Conversion between two conformational states of KaiC is induced by ATP hydrolysis as a trigger for cyanobacterial circadian oscillation. *Sci. Rep.* **6,** 32443 (2016).
12. Sugiyama, M. *et al.* Structural characterization of the circadian clock protein complex composed of KaiB and KaiC by inverse contrast-matching small-angle neutron scattering. *Sci. Rep.* **6,** 35567 (2016).
13. Snijder, J. *et al.* Structures of the cyanobacterial circadian oscillator frozen in a fully assembled state. *Science* **355,** 1181–1184 (2017).
14. Clodong, S. *et al.* Functioning and robustness of a bacterial circadian clock. *Mol. Syst. Biol.* **3,** 90 (2007).
