# Supplementary material for: Revealing circadian mechanisms of integration and resilience by visualizing clock proteins working in real time
Source: Nat Commun. 2018 Aug 14;9:3245. doi: 10.1038/s41467-018-05438-4 (PMC6092398; doi:10.1038/s41467-018-05438-4)
Supplement: Supplementary file 2 — Description of Additional Supplementary Files [file 41467_2018_5438_MOESM2_ESM.docx]

**Description of Additional Supplementary Files**

**File Name: Supplementary Movie 1**

**Description:**

Representative HS-AFM movie of KaiCI-WT interacting with KaiA. (Movie 1.mov)

**File Name: Supplementary Movie 2**

**Description:**

Representative HS-AFM movie of KaiCII-WT interacting with KaiA. (Movie 2.mov)

**File Name: Supplementary Movie 3**

**Description:**

Representative HS-AFM movie of KaiCI-DC plus KaiA. (Movie 3.mov)

**File Name: Supplementary Movie 4**

**Description:**

Representative HS-AFM movie of KaiCII-DC plus KaiA. (Movie 4.mov)

**File Name: Supplementary Movie 5**

**Description:**

Representative HS-AFM movie of KaiCI-WT in 2 mM ATP (without KaiA). (Movie

5.mov)

**File Name: Supplementary Movie 6**

**Description:**

Representative HS-AFM movie of KaiCII-WT in 2 mM ATP (without KaiA). (Movie

6.mov)

**File Name: Supplementary Movie 7**

**Description:**

Representative HS-AFM movie of KaiCI-AA in 2 mM ATP visualized at a frame rate of 1 fps (without KaiA). (Movie 7.mov)

**File Name: Supplementary Movie 8**

**Description:**

Representative HS-AFM movie of KaiCI-AA in 2 mM ATP visualized at a frame rate of 5 fps (without KaiA). (Movie 8.mov)

**File Name: Supplementary Movie 9**

**Description:**

Representative HS-AFM movie of KaiCI-WT (32% phosphorylated) in 2 mM ATP

visualized at a frame rate of 1 fps (without KaiA). (Movie 9.mov)

**File Name: Supplementary Movie 10**

**Description:**

Representative HS-AFM movie of KaiCI-WT (32% phosphorylated) in 2 mM ATP

visualized at a frame rate of 5 fps (without KaiA). (Movie 10.mov)

**File Name: Supplementary Movie 11**

**Description:**

Representative HS-AFM movie of KaiCI-DE in 2 mM ATP visualized at a frame rate

of 1 fps (without KaiA). (Movie 11.mov)

**File Name: Supplementary Movie 12**

**Description:**

Representative HS-AFM movie of KaiCI-DE in 2 mM ATP visualized at a frame rate

of 5 fps (without KaiA). (Movie 12.mov)

**File Name: Supplementary Movie 13**

**Description:**

Representative HS-AFM movie of KaiCII-DE interacting with KaiA. (Movie 13.mov)

**File Name: Supplementary Movie 14**

**Description:**

Representative HS-AFM movie of KaiCII-DA interacting with KaiA. (Movie 14.mov)

**File Name: Supplementary Movie 15**

**Description:**

Representative HS-AFM movie of KaiCII-AE interacting with KaiA. (Movie 15.mov)

**File Name: Supplementary Movie 16**

**Description:**

Representative HS-AFM movie of KaiCII-AA interacting with KaiA. (Movie 16.mov)

**File Name: Supplementary Movie 17**

**Description:**

Representative HS-AFM movie of the hyperphosphorylated KaiCII-WT (81%

phosphorylated) interacting with KaiA. (Movie 17.mov)

**File Name: Supplementary Movie 18**

**Description:**

Representative HS-AFM movie of the hypophosphorylated KaiCII-WT (27% phosphorylated) interacting with KaiA. (Movie 18.mov)
